# Pyrithione metal (Cu, Ni, Ru) complexes as photo-catalysts for styrene oxide production

**DOI:** 10.1038/s41598-021-03085-2

**Published:** 2021-12-10

**Authors:** Venkata D. B. C. Dasireddy, Jerneja Kladnik, Romana Cerc Korošec, Blaž Likozar, Iztok Turel

**Affiliations:** 1 Department of Catalysis and Chemical Reaction Engineering, National Institute of Chemistry Slovenia, Hajdrihova 19, 1001 Ljubljana, Slovenia; 2 Faculty of Chemistry and Chemical Technology, University of Ljubljana, Večna pot 113, 1000 Ljubljana, Slovenia

**Keywords:** Analytical chemistry, Catalysis, Photochemistry

## Abstract

Selective photochemical oxidation of styrene was performed in an active acetonitrile medium, using H_2_O_2_ with or without ultraviolet (UV) light radiation. Pyrithione metal complexes (M–Pth: M = Cu(II), Ni(II), Ru(II); Pth = 2-mercaptopyridine-*N*-oxide) were used as catalysts. Catalytic testing measurements were done by varying the time, chemical reaction temperature and H_2_O_2_ concentration with or without UV energy. Epoxide styrene oxide (SO), benzaldehyde and acetophenone were the major synthesized products. A high batch rate, conversion and selectivity towards SO was shown in the presence of UV. A minor constant formation of CO_2_ was observed in the stream. Coordinated Ru-based compounds demonstrated the highest process productivity of SO at 60 °C. The effect of the functional alkyl substituent on the ligand Pth, attached to the specific ruthenium(II) centre, decreased the activity of the substance. Ni-Pth selectively yielded benzaldehyde. The stability of the catalysts was examined by applying nuclear magnetic resonance (NMR) spectroscopy and thermogravimetric analysis coupled with mass spectrometry. Tested metal complexes with pyrithione (M–Pth) exhibited excellent reuse recyclability up to 3 cycles.

## Introduction

The oxidative transformation of hydrocarbons is an economical way for the production of corresponding alkenes^1,2^. Synthesis of oxides, epoxides, ketones and aldehydes through the catalytic oxidation of alkenes is a significant revolution in the organic transformation reactions of pharmaceutical industries^3–5^. Among the various epoxides synthesised through the catalytic oxidation of alkenes, styrene oxide was an important product used as a precursor for the synthesis of fine chemicals, plastics, and perfumery products. From the past decade, photocatalytic oxidation attracted the researchers' interest as the photocatalytic oxidation process is controllable by tuning the properties of the heterogeneous and homogenous catalyst and achieving a high yield of the targeted products^6–8^. The latest research works show that the photocatalytic process can offer a low-cost and green reaction pathway for the transformation of alkenes.

Chen et al.^8^ demonstrated the photocatalytic oxidation of alkenes and alcohols by [Mn^V^(N)(CN)_4_]^2–^ without any organic solvent. The results showed that the oxo species of Mn^V^(N) acts as an active oxidation species for photochemical oxidation catalysts. Catalytic selective oxygenation of cyclohexane by oxygen over [(TAML)Fe^III^]^−^ the complex was reported by Sankaralingam et al.^9^. The iron(III) complex with a tertraamido macrocyclic ligand showed an autocatalytic reaction mechanism for the selective catalytic oxidation of cyclohexane without a generation of free radical mechanism. Recently, Manrique et al.^10^ reported a new aqua complex *trans*‐[Ru^II^(trpy)(pypz‐H)(OH_2_)](PF_6_)_2_, as a catalyst in the photocatalytic oxidation of alkenes like 1-octene and styrene, and it exhibited conversions > 50%. The [Ru^v^ = O]^2+^ acted as an active species in the complex, which showed moderate to high conversions and good selectivity values for the epoxides. Similarly, Kalita et al.^6^ also showed that Ru^v^ = O species in [Ru^II^(bpy)_3_]Cl_2_ has been confirmed as a highly active species for the photocatalytic oxygenation of hydrocarbons. Literature data thus suggest that also metal(II) complexes can be promising catalysts for a highly active photochemical oxidation of alkenes^11–14^.

**Figure 1:**
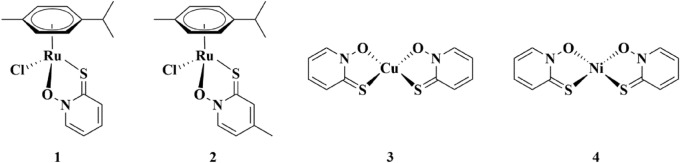
Structures of tested metal catalysts 1) Ru-Pth, 2) Ru-Pth-Me, 3) Cu-Pth and 4) Ni-Pth.

This study focused on various metal complexes, namely Ru^II^, Cu^II^ and Ni^II^, with the ligand 2-mercaptopyridine-*N*-oxide or pyrithione (Pth) (Fig. 1). Pyrithione in its deprotonated form binds to metal ions via *O-* and *S-*atoms and its complexes are reported to have good biological activity. Turel group has done extended research on organoruthenium(II)-pyrithionato complexes, which are biologically active and possess anticancer activity^15–18^. Nickel pyrithione (Ni-Pth) was described to have antileukemic effect^19,20^, whereas copper pyrithione (Cu-Pth) is widely used as a biocide in commercially available antifouling paints^21^. Besides, Ni-Pth also turned out to be a potent catalyst for light-driven and electrocatalytic hydrogen production from water^11^. To the best of our knowledge mentioned complexes have not been studied in any photocatalytic oxidation of styrene to styrene oxide. Considering all the above-mentioned necessities for the production of styrene oxide and finding new catalysts for the selective oxidation of styrene, we have decided to explore the potential of pyrithionato complexes in this system.

## Results and discussion

Ruthenium complexes Ru-Pth and Ru-Pth-Me were prepared according to^16^. Ruthenium precursor [Ru(*p*-cymene)Cl_2_]_2_, appropriate ligand either Pth or its methyl-substituted derivate Pth-Me (1-hydroxy-4-methylpyridine-2(1*H*)-thione) and the base NaOMe to deprotonate pyrithiones were stirred in the chosen solvent overnight at 20 °C. After the purification of the complexes by column chromatography, the red solid was obtained by precipitation from DCM/heptane solvent system. Copper Cu-Pth and nickel Ni-Pth complexes, investigated here, were prepared according to the modified procedure for similar zinc complexes^22^. However, there have also been similar procedures reported for copper^23^ and nickel pyrithione complexes^12^. In the case of nickel and copper complexes, Pth was first dissolved in methanol. With the addition of NaOH, the neutral ligand was converted to sodium salt, which easily reacts with the chosen metal salts, namely copper(II) and nickel(II). After the addition of CuCl_2_·2H_2_O copper complex immediately precipitated out, whereas prolonged reaction time in case of the addition of Ni(CH_3_CO_2_)_2_·4H_2_O was needed for the reaction to be completed. The precipitated green cooper and dark red nickel complexes were filtered under reduced pressure and washed with methanol to get rid of the NaCl or CH_3_COONa and later additionally with diethyl ether. Previously reported crystal structures are shown in Fig. 1, together with IR spectra of the ligands, ruthenium precursor and metal complexes (Electronic Supplementary Information, Figures S5–12).

**Figure 2:**
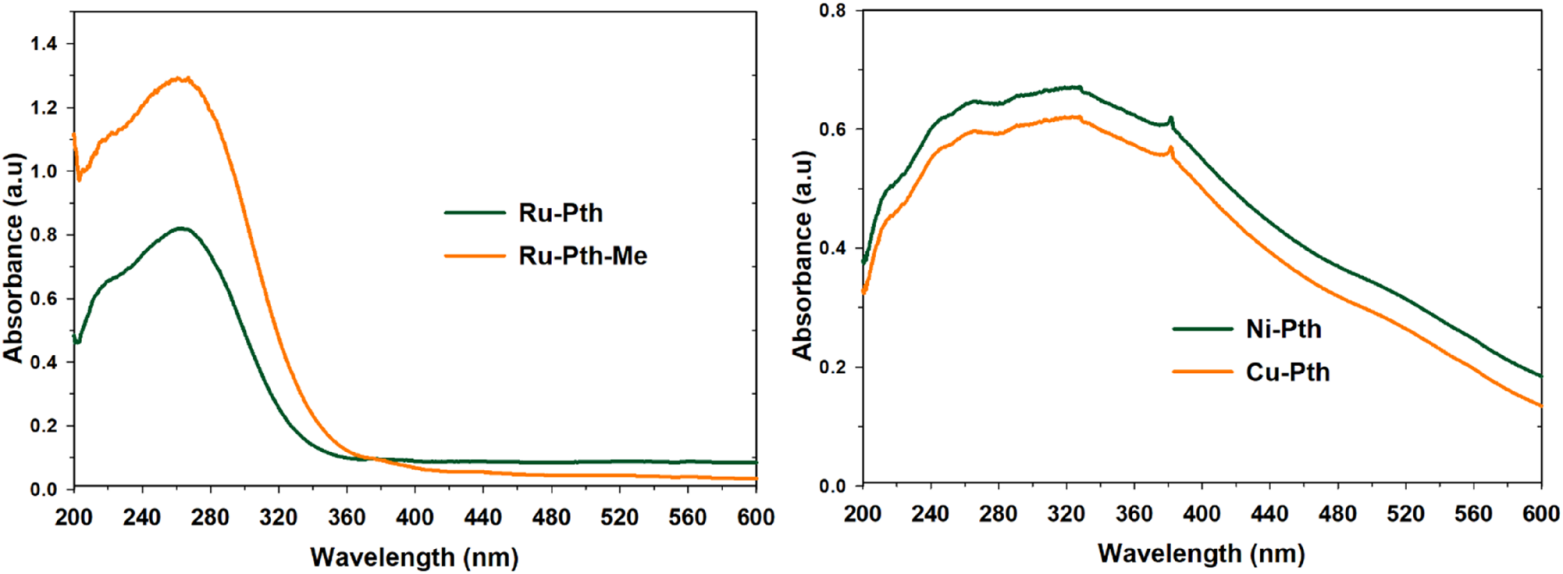
Diffuse reflectance UV–Vis spectra of Ru-Pth, Ru-Pth-Me, Ni-Pth and Cu-Pth complexes.

The UV–Vis optical absorption spectra of the prepared complexes are shown in Fig. 2. Both Ru complexes exhibited higher absorbance when compared to Ni and Cu complexes. The main peak for all the complexes at around 260 nm corresponds to the π–π* plasmon peak and a broad peak around 310 nm corresponds to the n–π* plasmon peak. Both Ru complexes showed the π–π* plasmon peak and the other Ni and Cu complexes showed the n–π* plasmon peaks. The presence of *O*- and *S*-atoms in the complexes can lead to the enhancement of light absorbance^24^. Thus, in the present complexes *O*- and *S*-bonded ligands to the metal could have acted as electron carriers to transfer electrons generated from the metal to its conduction band. The bandgap energy of samples was determined using Kubelka–Munk Model^25^, by the equation Eg = 1239.8/ƛ where Eg is the bandgap energy (eV) and ƛ is the wavelength (nm) of the absorption edges in the spectrum (Electronic Supplementary Information, Figure S13). All Ru-based complexes showed bandgap energy in the 2.4–2.6 eV range, which agrees with the literature^26^. Ni and Cu complexes showed a higher bandgap 3.6–4.1 eV in comparison with Ru complexes. This could be due to Ru complexes' higher adsorption capacity, which various researchers in the literature^27,28,37,38^ also demonstrated.

**Figure 3:**
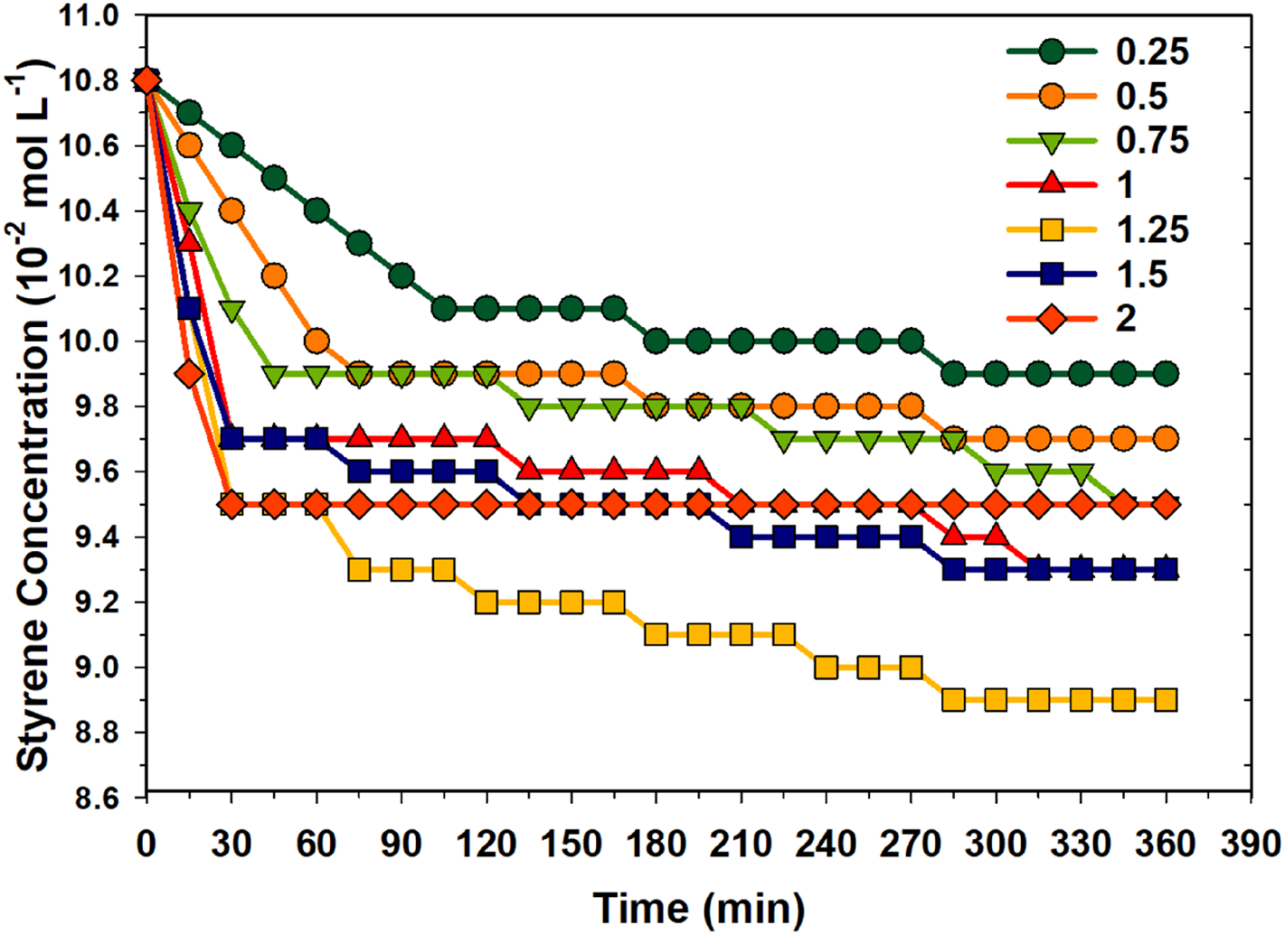
Change in styrene concentration at various H_2_O_2_ concentrations (10^–2^ mol L^−1^) as a function of time under UV radiation at STP conditions (without catalyst complexes). Reaction conditions: styrene: 10 mmol L^–1^; acetonitrile: 50 mL.

To investigate the catalytic activity of the complexes and the role of UV radiation on the formation of styrene oxide, firstly, the blank catalytic testing (without catalyst) was done under UV radiation and without UV radiation. No change in the concentration of styrene was observed without UV radiation. There was no effect of H_2_O_2_ concentration on the conversion of styrene without UV radiation. Figure 3 shows the change in the concentration of styrene under UV radiation while varying H_2_O_2_ concentration. It could be observed that as the concentration of H_2_O_2_ increased, the conversion of styrene also increased. As the time of the reaction increases, the concentration of styrene in the reaction mixture decreases linearly. After 120 min, at an H_2_O_2_ concentration of 1.25 10^–2^ mol L^−1^, a high decrease in styrene concentration was observed. At higher H_2_O_2_ concentrations, the generated oxygen species may be utilised for the secondary oxidation of formed products, thus resulting in a non-availability of oxygen radicals for the styrene conversion.

**Figure 4:**
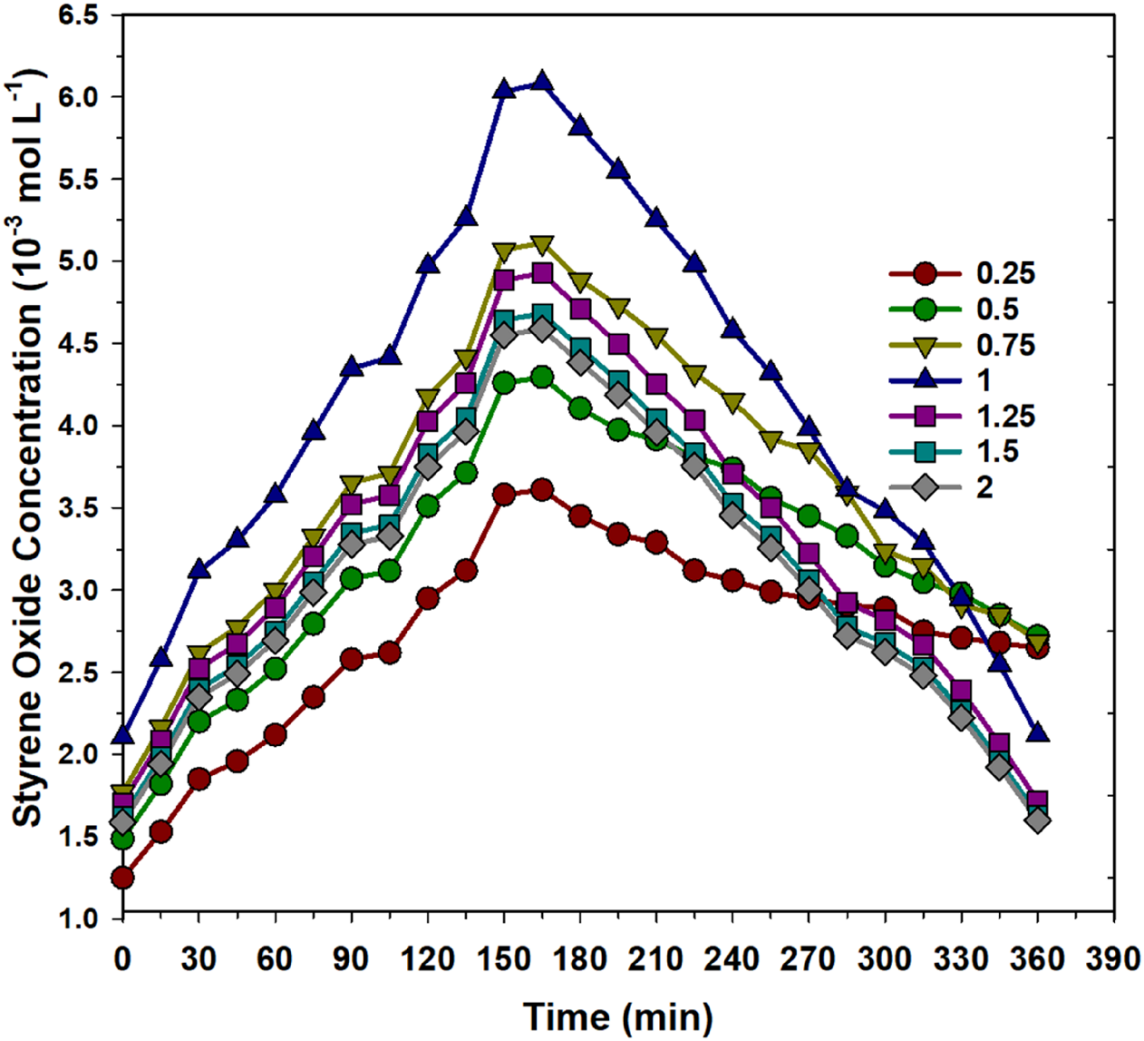
Concentration of styrene oxide at various H_2_O_2_ concentrations (10^–2^ mol L^−1^) as a function of time under UV radiation at STP conditions (without catalyst complexes). Reaction conditions: styrene: 10 mmol L^–1^; acetonitrile: 50 mL.

Figure 4 shows the effect of H_2_O_2_ concentration on the formation of styrene oxide as the function of time. As time increases, the styrene oxide formation increases up to 180 min, and the further increase in the time results in a decrease in the formation of styrene oxide. This could be due to the second conversion of styrene oxide to acetophenone. On the other hand, the styrene oxide formation increased up to an H_2_O_2_ concentration of 1 (10^–2^ mol L^−1^), and a further increase in the concentration of H_2_O_2_ resulted in a low selectivity towards styrene oxide. This agrees with the trend of the change in the styrene concentration as a function of time. At higher concentrations of H_2_O_2_, the secondary oxidation of styrene oxide proceeds at a much higher rate than that of styrene. This resulted in a decrease in the concentration of styrene oxide and an increase in styrene concentration. The highest styrene oxide was observed at the styrene to H_2_O_2_ molar ratio of 1 at 180 min. Thus, further catalytic testing was done at styrene to H_2_O_2_ molar ratio of 1 at 180 min. Although the amount of styrene oxide formed during the reaction was high, the conversion of styrene was low compared to the other non-photocatalytic reaction systems reported in the literature. Thus, the reaction temperature was increased from RT to 80 °C, and the change of the styrene conversion and selectivity of styrene oxide was examined.

**Figure 5:**
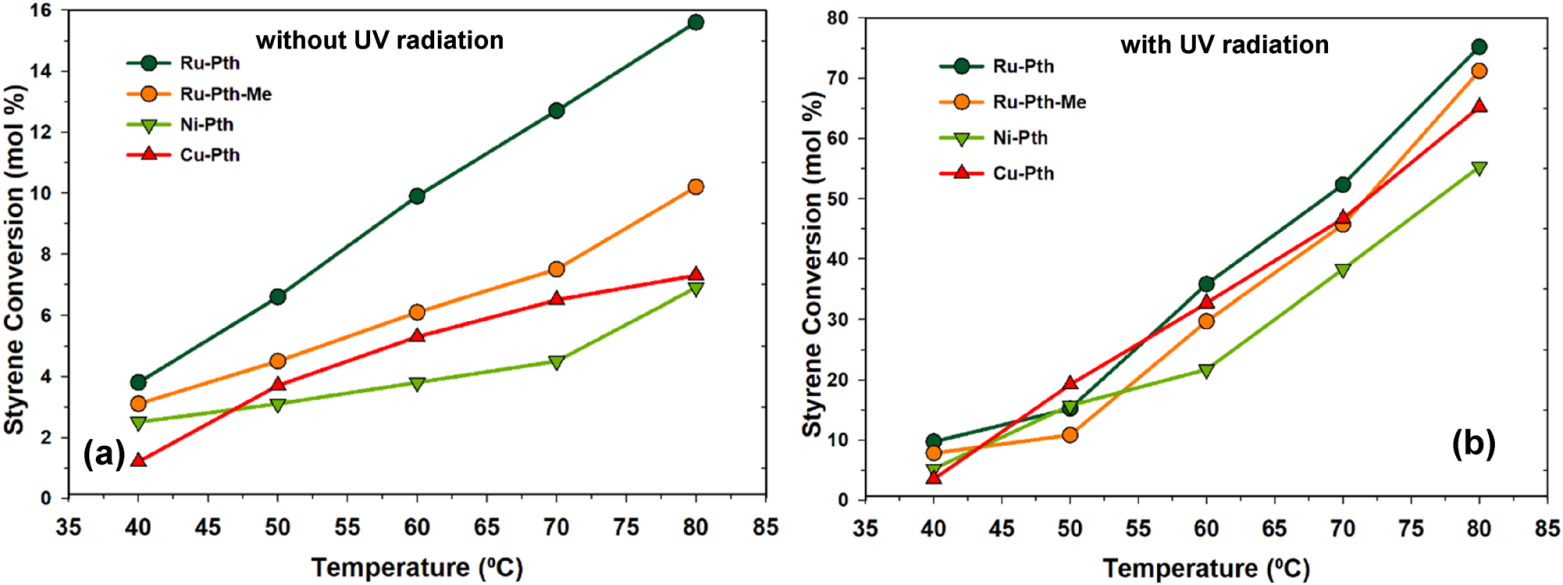
Effect of the temperature on the styrene conversion over Ru-Pth, Ru-Pth-Me, Ni-Pth and Cu-Pth complexes (**a**) without and (**b**) with UV radiation. Reaction conditions: catalyst: styrene (1:100); styrene to H_2_O_2_ ratio of 1; acetonitrile: 50 mL; catalyst: 10 mg; reaction time: 3 h.

**Figure 6:**
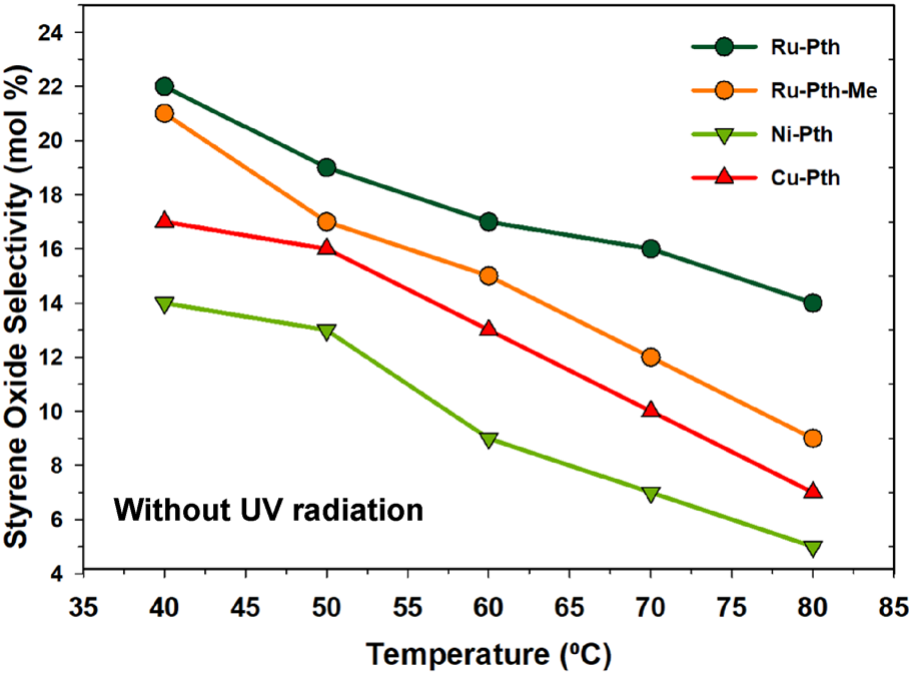
Effect of the temperature on the styrene oxide selectivity over Ru-Pth, Ru-Pth-Me, Ni-Pth and Cu-Pth complexes without UV radiation. Reaction conditions: catalyst: styrene (1:100); styrene to H_2_O_2_ ratio of 1; acetonitrile: 50 mL; reaction time: 3 h.

Figure 5a shows the effect of conversion on styrene conversion without UV radiation in the presence of metal-pyrithione complexes. It could be observed that as the temperature increases, the conversion of styrene increases, too. Among all catalysts, Ru-Pth and Ru-Pth-Me showed the highest styrene conversion. The conversion of styrene was very low compared to the other Ru catalytic systems reported in the literature. Figure 5b shows the effect of the temperature on styrene conversion under UV radiation. Similar to the one without UV radiation experiments, the conversion of styrene increased with increased temperature. Ru-Pth and Ru-Pth-Me catalysts showed high conversion in the presence of UV radiation again. The conversion of styrene increased from 16 to 75% in the presence of Ru-Pth at 80 °C due to the UV radiation exposure of the reaction mixture. All catalysts showed a conversion of styrene with a standard deviation of ± 5%, at lower temperatures (< 60 °C) and the gradient of difference in the conversion of styrene was bigger at higher temperatures (> 60 °C). There was no major change in the styrene concentration with the exposure of the photocatalytic radiation only. The combination of photolysis radiation and H_2_O_2_ with metal catalysts resulted in the high conversion of styrene (Fig. 5b). This suggests that the photolysis radiation enhances the oxidation of H_2_O_2,_ resulting in the formation of two hydroxyl radicals and cleavage of the O–O bond of H_2_O_2_. Figure 6 shows the effect of the temperature on the selectivity towards styrene oxide without the photolysis radiation. The selectivity towards styrene oxide decreased with an increase in temperature. This could be due to the secondary oxidation of styrene oxide and the formation of benzaldehyde due to the C=C bond cleavage of styrene. A low selectivity towards styrene oxide suggests that the H_2_O_2_ may promoted the C=C bond cleavage to form benzaldehyde or overoxidation of styrene oxide to acetophenone. In general, without the photolysis radiation, benzaldehyde can also be formed by the nucleophilic attack of H_2_O_2_ on the styrene oxide and the oxidative cleavage of the hydroperoxystyrene. On the other hand, without photolysis radiation, the higher oxidation potential of OH* (E^0^ = 2.8 eV) results in a very low conversion of styrene to styrene oxide, resulting in a very low selectivity towards styrene oxide.

**Figure 7:**
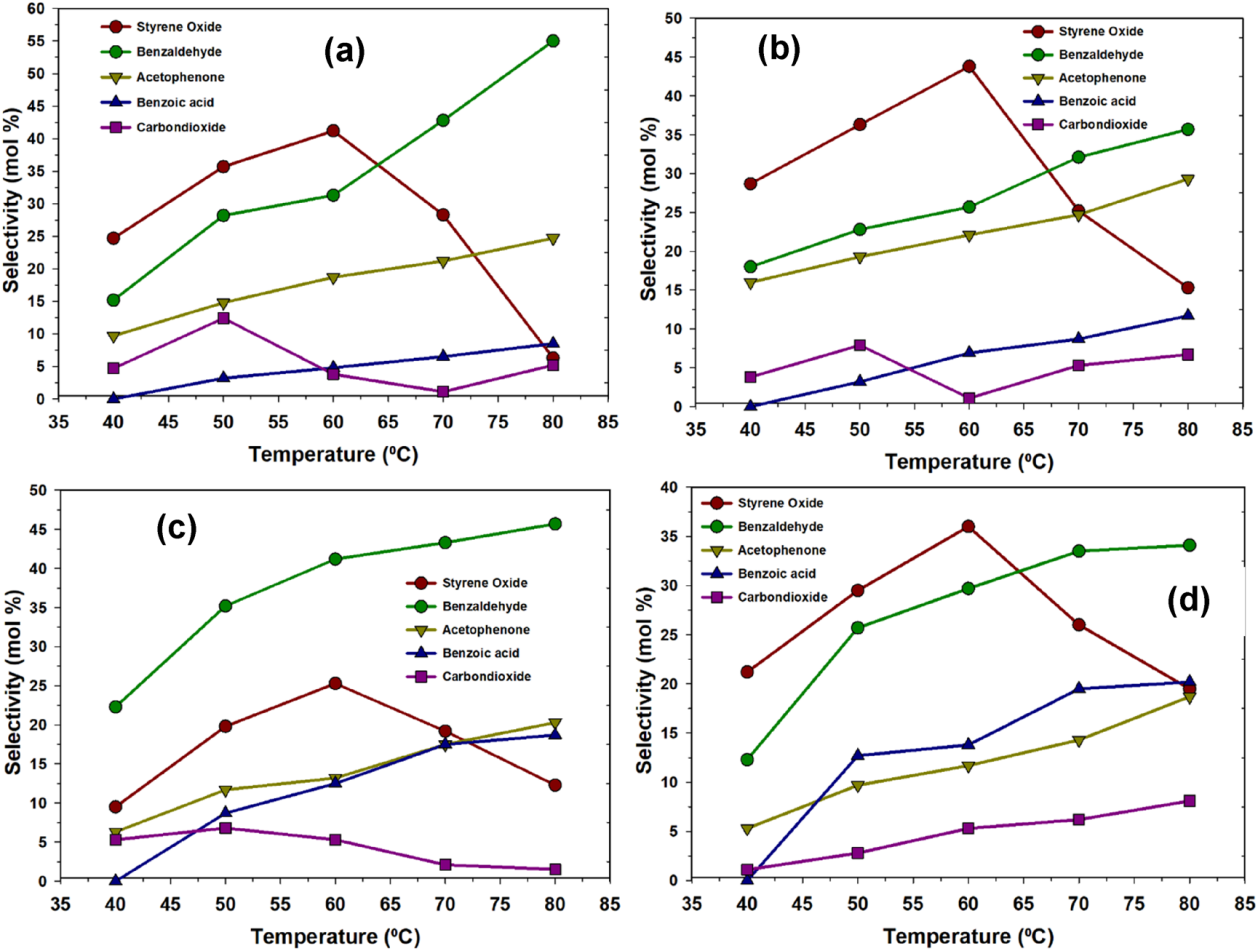
Effect of the temperature on the selectivity towards products in the oxidation of styrene over (**a**) Ru-Pth, (**b**) Ru-Pth-Me, (**c**) Ni-Pth and (**d**) Cu-Pth complexes under UV radiation. Reaction conditions: catalyst: styrene (1:100); styrene to H_2_O_2_ ratio of 1; acetonitrile: 50 ml; reaction time: 3 h.

Figure 7 shows the effect of the temperature on the on the selectivity towards products in the presence of photolysis radiation after 180 min. The highest selectivity towards styrene oxide was exhibited by Ru-Pth and Ru-Pth-Me catalyst at the temperature of 60 °C. As the temperature increases, the selectivity towards styrene oxide decreases, which could be due to the secondary oxidation of styrene oxide or the oxidative cleavage of styrene oxide. In the presence of the photolysis radiation, H_2_O_2_ tends to generate the electrophilic oxygen species as the oxidation potential of H_2_O_2_ (E^0^ = 1.78 eV) was low compared to the oxidation potential of OH*. In the presence of photolysis radiation, the decomposition of H_2_O_2_ leads to the generation of electrophilic oxygen species that are selective in the oxygen insertion reactions. Without the photolysis radiation, the decomposition of H_2_O_2_ generates the OH* active in the free radical mechanisms.

**Figure 8:**
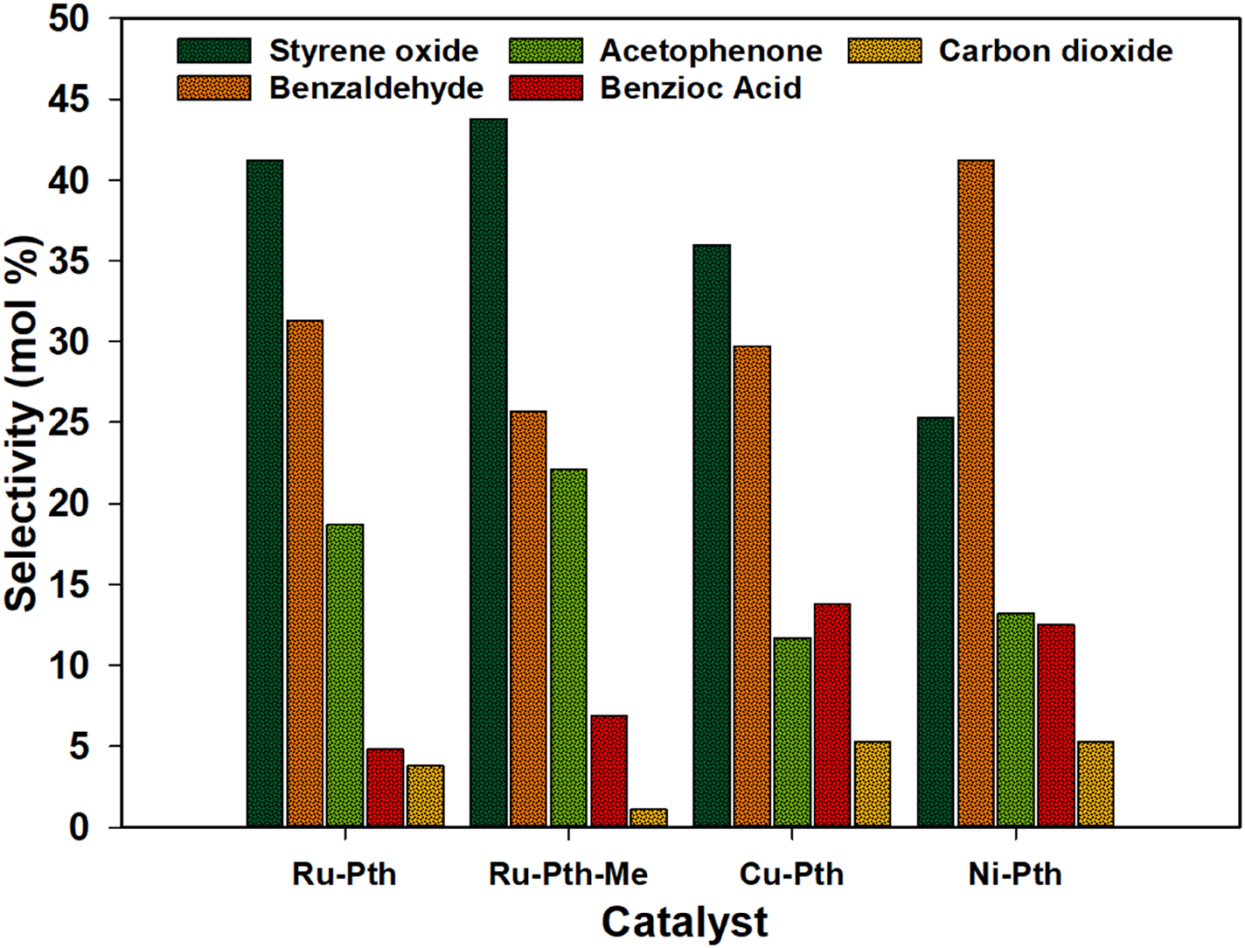
Selectivity towards products in the oxidation of styrene over various complexes under conditions: catalyst: styrene (1:100); styrene : H_2_O_2_ (1:1); solvent: acetonitrile; temperature: 60 °C.

Among all catalysts, Ru complexes showed high selectivity towards styrene oxide, which could be due to the lower affinity of Ni^II^ and Cu^II^-oxo complexes towards olefins. The highest selectivity is exhibited by Ru-Pth and Ru-Pth-Me complexes at 60 °C after 180 min (Figs. 7, 8). High activity of Ru-Pth and Ru-Pth-Me complexes could be due to the chlorido group coordinating to the Ru-atom^29^. The difference in the activity of the catalysts in the styrene oxidation could be attributed to different substituents, which results in the basicity of the catalyst. The presence of *N*-atom and substituent on the *N*-atom direct the basicity of the catalyst, and if the basicity increases, the activity of the catalyst decreases. In all cases of the catalysts, the *N*-atom was bonded to *O*-atom, which was further bonded to the metal; thus, the basicity of the material is due to the presence of metal compared to the substituent of *N*- atom. Puddephatt et al.^30^ showed that pyridine or tertiary amine *N*-oxide derivates could act as oxygen atom donors with hard metal ions and prefer C–H bond activation over N–O bond activation. In general, the basic substituent in the catalyst increases the electron density at the metal centre, which makes it easier for the oxidation process to occur. However, this increases the secondary oxidation/cleavage of styrene oxide and decreases the selectivity towards styrene oxide. In general, the electron-withdrawing substituents are more active when compared to the alkyl substituents^31^. Thus, the presence of alkyl substituent in Ru-Pth-Me resulted in the lower activity of the catalyst than the Ru-Pth catalyst without any alkyl substituent (Table 1). Similarly, high selectivity towards styrene oxide was observed over Ru^III^ triphenylphosphine complexes^32^. These Ru complexes also showed much lower bandgap energy in comparison with Ni and Cu complexes. The majority of the metal complexes have larger band gaps (Eg > 3 eV), which agrees with the Cu and Ni complexes. Ru-pth and Ru-Pth-Me complexes showed lower band gaps (< 3 eV), enhancing photocatalytic activity in styrene oxidation.

**Table 1:** Selective photocatalytic oxidation of styrene-to-styrene oxide over metal pyrithione complexes.

Catalyst	Styrene conversion (%)	Styrene oxide selectivity (mol%)	Yield of styrene oxide (mol%)	Turnover frequency (10^–2^ s^−1^)	Turnover number
Ru-Pth	35.8	41.2	14.8	1.9	436
Ru-Pth-Me	29.7	43.8	13.0	1.7	464
Ni-Pth	21.7	25.3	9.1	0.9	268
Cu-Pth	32.7	36.0	10.7	1.1	381

Reaction conditions: Catalyst to styrene ratio of (1:100); styrene : H_2_O_2_ (1:1); time: 3 h; solvent: acetonitrile; temperature: 60 °C, Turnover frequency (TOF) = moles of styrene converted/(moles of metal × time); Turnover number (TON) = moles of styrene oxide formed/moles of metal.

To examine the activity of the catalyst, the selectivity towards the products were determined at an iso-conversion (100% conversion of styrene) at a temperature of 60 °C (Fig. 8). The product distribution agrees with the selectivity pattern obtained earlier, i.e., Ni-Pth complex showed high selectivity towards benzaldehyde and the other complexes Ru-Pth, Ru-Pth-Me and Cu-Pth showed high selectivity towards styrene oxide. To determine the mechanism of the reaction, the reactions were conducted using styrene oxide and benzaldehyde as a substrate over Ru-complexes (Electronic Supplementary Information, Tables S1 and S2). Benzaldehyde, benzoic acid and acetophenone were the products when styrene oxide was used as a substrate. Benzoic acid was the only product observed with the benzaldehyde substrate. This suggests that the formation of benzaldehyde occurs through two pathways (1) C=C cleavage of styrene and (2) C=C bond cleavage of styrene oxide. There is only one pathway for the formation of styrene oxide, i.e. electrophilic attack of H_2_O_2_ on styrene. In general, three chemical pathways are proposed in the conversion of styrene with two HO_2_° radicals, or one HO° and O_2_°- or one HO_2_° and one O_2_°. The mechanism is based on the active species formed by the decomposition of H_2_O_2_ (and H_2_O) and in presence of of photocatalytic radiation.

Table 1 shows the turnover frequency of styrene conversion and the turnover number of styrene oxide formation over the metal pyrithione complexes. The turnover numbers and turnover frequencies were comparable to the other heterogeneous catalytic systems reported in the literature. Yajie et al.^33^ showed high selectivity towards styrene oxide (82%) over Au-Ti-SBA-15 mesoporous catalysts under photocatalytic conditions. A high TOF of styrene (0.79 10^–2^ s^−1^) was obtained in the presence of basic pH conditions. A similar observation was done by Hinda et al.^34^, using TiO_2_ and ZnO as catalysts. At basic pH, the conversion of styrene increased and a 50% selectivity towards styrene oxide was obtained. In general, the basic pH of the reaction medium favours high selectivity towards styrene oxide. Numerous works reported using base (NaOH or KOH) to control the pH of the reaction medium for the photocatalytic oxidation reactions^35–37^. In the present study, metal pyrithione complexes showed a high conversion of styrene and a high selectivity of styrene oxide without any addition of NaOH to the reaction medium. A high TOF of styrene conversion and turnover number of styrene oxide was obtained when compared to polystyrene carbon coated titania^38^, TiO_2_/H_2_O_2_ and ZnO/H_2_O_2_ catalytic systems^34^.

**Figure 9:**
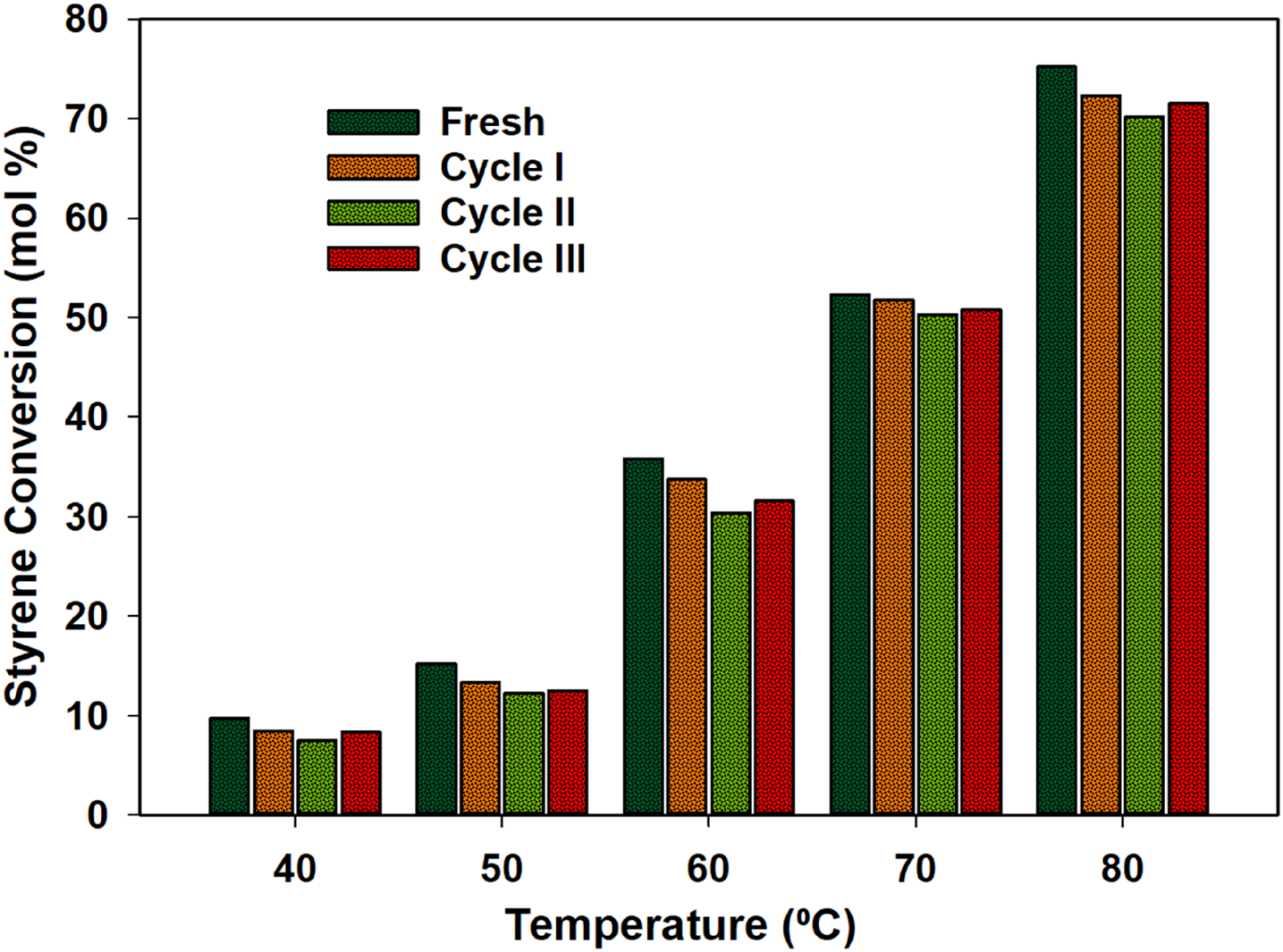
Conversion of styrene over the temperature over the Ru-Pth catalyst under conditions: catalyst: styrene (1:100); styrene: H_2_O_2_ (1:1); time: 3 h, solvent: acetonitrile.

One of the important features of the catalytic system that we have used was the recovery of the catalyst. In general, the *N*- and *S*-based complexes are oxidized due to the presence of H_2_O_2_. After the reaction, the catalysts were recovered and characterised using NMR, where the spectra were compared to those of the fresh catalysts. All catalysts were recovered and used for the recycle tests. After the recovery, the catalysts were subjected to similar reaction conditions as the fresh catalysts. There were no major differences in the conversion of styrene and selectivity towards the products. The styrene conversion and styrene oxide selectivity were in the range of ± 1% for all recycle tests (Fig. 9). The catalysts showed excellent stability and activity up to 3 cycles.

## NMR and TG stability

**Figure 10:**
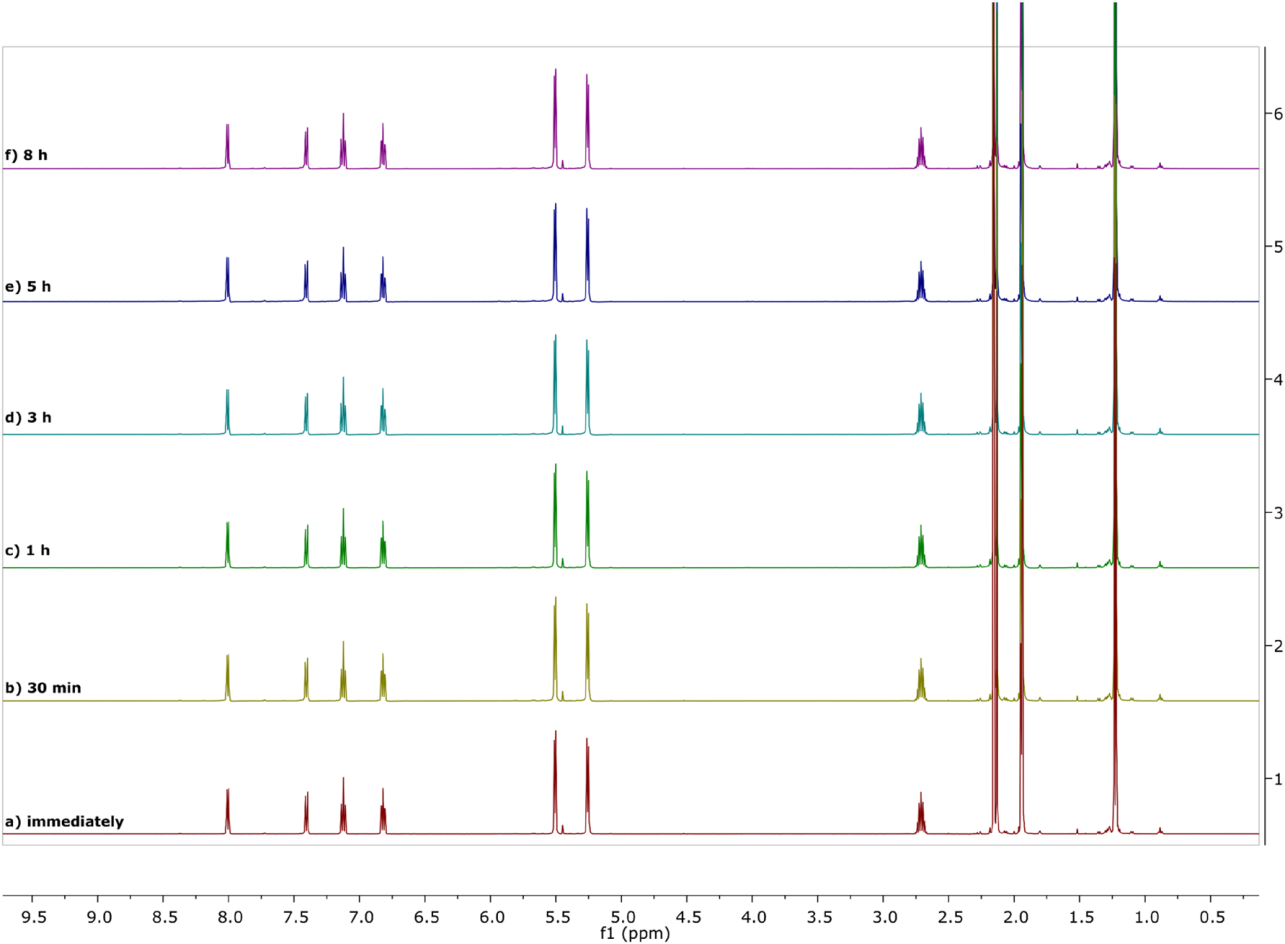
Spectra of Ru-Pth in acetonitrile-d_3_ followed by ^1^H NMR spectroscopy at different time points.

To check whether the compounds remain stable during the process, NMR experiments (before and after the photocatalytic reaction) were run for Ru-Pth, Ru-Pth-Me and Ni-Pth, but not for Cu-Pth as it is paramagnetic. Before performing the photocatalytic experiments, we have followed the stability of both ruthenium and nickel complexes in acetonitrile-d_3_ as this (non-deuterated) solvent was also used in the catalytic runs. The spectra were recorded immediately after the preparation and then later at the chosen time points. Figure 10 shows that Ru-Pth is a stable catalyst throughout the 8-h NMR reactivity tracking. It can be observed that the Ru-Pth-Me catalyst also remains stable during the timeframe used for following the stability (Electronic supplementary information, Figure S14). The negligible release of *p*-cymene ring from ruthenium(II) centre can be observed first after 8 h. Also, Ni-Pth is stable in acetonitrile-d_3_ solution over the observed time (Electronic supplementary information, Figure S15). Considering that under optimized conditions, the catalytic runs last 3 h, we can conclude that initially used metal compounds are responsible for their photocatalytic activity.

A comparison of the thermal stability of all four synthesized complexes is presented in Electronic Supplementary Information, Figure S16. Both organoruthenium complexes, Ru-Pth and Ru-Pth-Me, are less thermally stable than copper and nickel pyrithione complexes. The thermal stability of the former two is similar; thermal decomposition starts around 155 °C while Cu-Pth and Ni-Pth complex decomposition begins at 100° higher temperature, around 265 °C. These two complexes also possess similar thermal stability. Therefore, the tested complexes display relatively high thermal stability. According to TG analysis, the highest temperature used for investigating photocatalytic activity was 80 °C, the heating should not affect the catalysts' structures.

**Figure 11:**
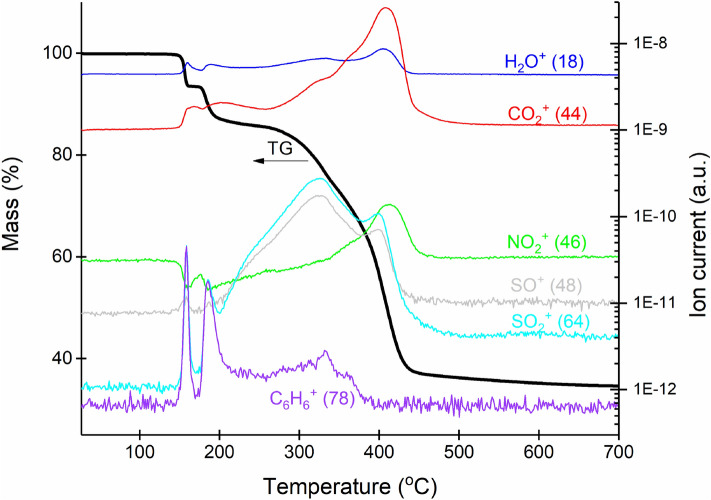
TG-MS curves of Ru-Pth complex (black line—TG curve, other lines—different m/z peaks).

Further, thermal decomposition of the photocatalytically most efficient ruthenium complex Ru-Pth was investigated using thermogravimetry, coupled with mass spectroscopy (Fig. 11). The complex stays thermally stable up to approximately 150 °C and then decomposes in four successive steps. From the intensity of the signal 78 m/z during first decomposition step (this signal is typical for benzene ring), we assume that decomposition of this complex starts with the breaking of a π-bond between ruthenium and *p*-cymene ring, and this could be a possible reason for lower thermal stability of both ruthenium complexes. The molecular peak for *p*-cymene 134 m/z was not detected because only the signals in the range from 15 to 90 m/z were collected on the mass spectrometer. We have noticed that heavier fragments are not detected, most probably due to (1) electron ionization and consequent fragmentation of higher species and (2) their condensation in the capillary, which connects the exit of the furnace with the mass spectrometer. In addition to 78 m/z signal, which was chosen from the large group of the signals present in the mass spectrum of *p*-cymene^39^, the following signals were also detected in the mass spectrometer: 18, 44, 46, 48 and 64. They most probably belong to the water, CO_2_, NO_2_, SO and SO_2_, respectively. Since the intensity of the last three (NO_2_, SO and SO_2_) becomes pronounced after 200 °C, we assume that pyrithionato ligands mostly decompose in the last two steps.

**Figure 12:**
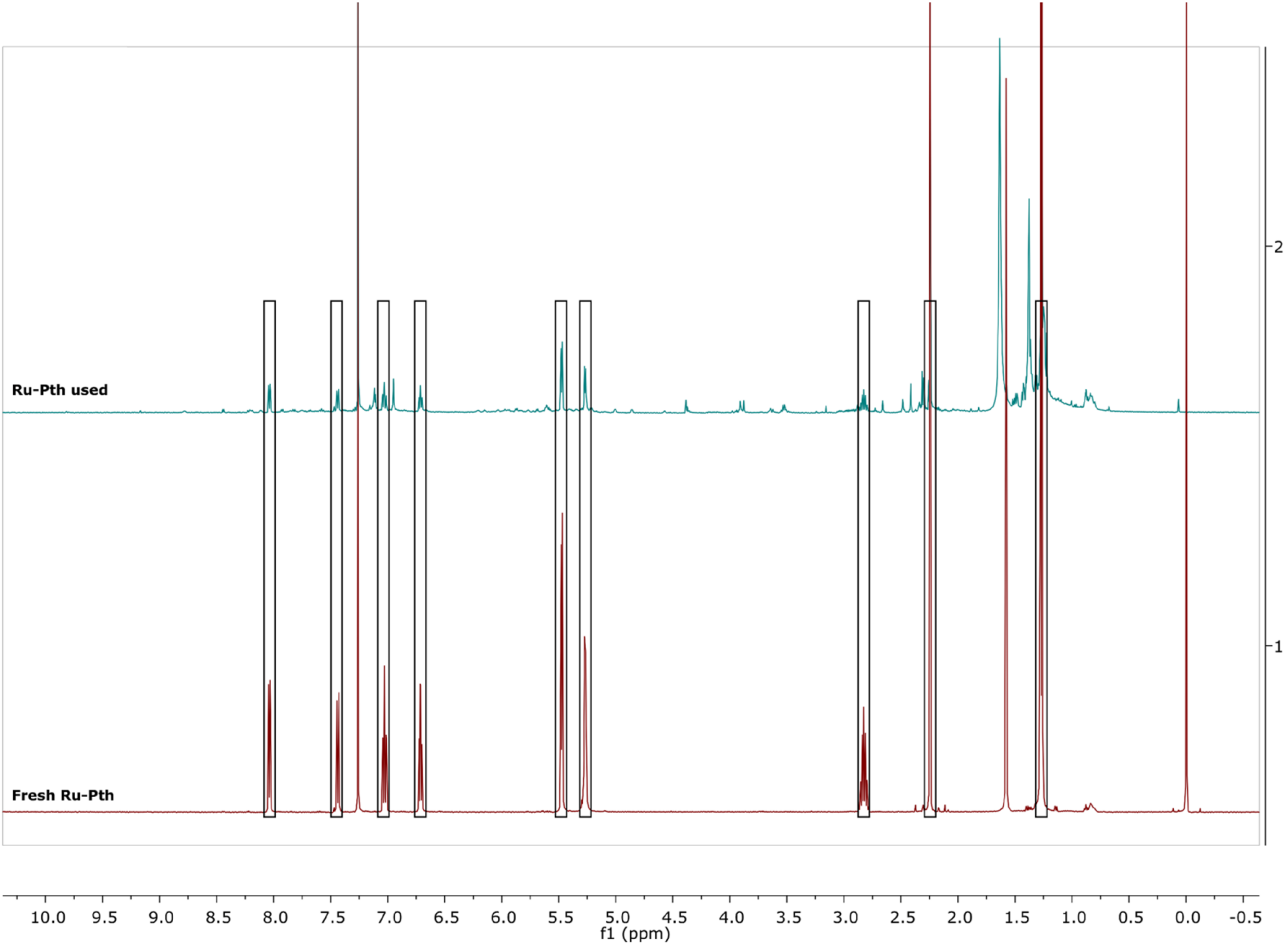
^1^H NMR spectra in CDCl_3_ of the initial Ru-Pth complex before photocatalysis (fresh Ru-Pth) and after (Ru-Pth used) under optimized conditions (catalyst: styrene (1:100); styrene : H_2_O_2_ (1:1); time: 3 h; solvent: acetonitrile; temperature: 70 °C. The additional peaks in the spectrum of Ru-Pth used occur as the crude reaction mixture after photocatalysis was recorded.

Later, the stability of the compounds Ru-Pth, Ru-Pth-Me and Ni-Pth were proved after the photocatalysis was performed. The catalysts used were recovered, ^1^H NMR spectra were obtained and compared to the spectra of the fresh catalysts before catalytic runs (Fig. 12). From comparing the initial spectra of the complexes recorded before and after catalysis, we can conclude that the tested compounds remain stable during the catalytic conditions used as the proton peaks overlap in all cases (Fig. 12; Electronic supplementary information, Figures S17, 18).

The photocatalytic oxidation of styrene was successfully performed with metal (Ru, Cu and Ni) pyrithione complexes. These catalysts showed excellent stability with a high yield conversion towards styrene oxide. The catalytic results showed that (Ru, Cu and Ni) pyrithione complexes promoted the photolysis of hydrogen peroxide, which further increased the formation of styrene oxide. In the present study, metal pyrithione complexes showed a high conversion of styrene and high styrene oxide selectivity without adding NaOH or any other base to the reaction medium. The highest selectivity of styrene oxide (> 40%) at 180 min and 60 °C was exhibited by Ru-Pth and Ru-Pth-Me which could be due to the presence of both Ru–O and the chlorido group coordinated to the Ru atom. The difference in the activity of the catalysts in the styrene oxidation could be attributed to the different substituents, which results in the basicity of the catalyst. Ru-Pth complex showed a TOF of 1.9 10^–2^ s^−1^ and a high styrene oxide TON of 464 was shown by Ru-Pth-Me complex. A high TOF of styrene conversion and turnover number of styrene oxide was obtained over metal pyrithione complexes compared to various photocatalytic systems reported in the literature.

## Materials and methods

Ligand pyrithione or 2-mercaptopyridine-*N*-oxide (99%, Fluorochem), starting materials for the synthesis of the ligand for Ru-Pth-Me complex, ruthenium precursor [(*p*-cymene)RuCl_2_]_2_ (min. 98%, Strem Chemicals), CuCl_2_·2H_2_O (Riedel-de Haën), Ni(CH_3_CO_2_)_2_·4H_2_O (98%, Fluorochem) were purchased from commercial suppliers and used as received. Solvents (Honeywell) were used as p.a. grade, except from methanol which was gradient grade and used without further purification. To follow the progress of the reactions pre-coated TLC sheets ALUGRAM^®^ SIL G/UV_254_ (Macherey–Nagel) were used and visualized under UV light. Column chromatography was performed with a stationary phase with Merck Silica gel 60 (35–70 µm). ^1^H NMR spectra were recorded at 500 MHz NMR using Bruker Avance III 500 spectrometer at room temperature. Chemical shifts are referenced to deuterated solvent residual peaks of CDCl_3_ and CD_3_CN at 7.26 ppm and 1.94 ppm (the central line of a quintet), respectively. Chemical shifts (*δ*) and coupling constants (*J*) are given in ppm and Hz, respectively. MestReNova version 11.0.4 was used for NMR data processing. Infrared spectra were recorded with a Bruker FTIR Alpha Platinum ATR spectrometer. High-resolution mass spectra (HRMS) were recorded on an Agilent 6224 Accurate Mass TOF LC/MS instrument. Elemental analyses were carried out on a Perkin-Elmer 2400 II instrument (CHN). The diffuse reflectance UV–Vis spectra of catalysts were recorded in the 200–900 nm region using the PerkinElmer Lambda 40 Spectrometer. All data were recorded after 5 cycles (averaging five measurements) with the interval of 1 nm, slit width of 2 nm, the scan speed of 10 nm min^−1^, and spectral resolution of 0.5 nm. The thermal stability of the prepared complexes was determined from the results of thermogravimetric measurements performed on a Mettler Toledo TGA/DSC1 instrument. For the measurement, cca 5 mg of the investigated sample was placed into 150 µL platinum crucibles and heated from 25 to 400 °C with a heating rate of 10 K min^‒1^. During the measurement, the furnace was purged with air with a flow rate of 50 mL min^‒1^. The blank curve was subtracted. Coupled TG-MS measurement of the most photocatalytically active sample (Ru-Pth) was performed on the same TGA instrument under the same conditions as previously described in a temperature range from room temperature to 700 °C. Evolved gases were introduced into the mass spectrometer via 75-cm long heated capillary (T = 190 °C), connected to a Pfeiffer Vacuum ThermoStar mass spectrometer.

### Syntheses and characterization

Ru-Pth and its methyl derivate Ru-Pth-Me were synthesized according to the previously published procedure reported by Turel group^16^. Cu-Pth and Ni-Pth were prepared according to the previously reported synthesis for similar zinc complexes^22^ with modifications described below. The physicochemical characterization of prepared compounds was performed by ^1^H NMR and infrared (IR) spectroscopy, CHN elemental analysis and high-resolution electrospray ionization mass spectrometry (ESI-HRMS). The purity of synthesized compounds was confirmed using NMR spectroscopy (except for the copper complex, a paramagnetic compound) and CHN elemental analysis.

### Bis[1-hydroxypyridine-2(1***H***)-thionato-***S,O***]copper(II) (Cu-Pth)

Ligand pyrithione (also 2-mercaptopyridine-*N*-oxide; 70 mg, 2 mol. equiv.) was dissolved in 5 mL of methanol. While stirring 1 M NaOH_(aq)_ was added dropwise to reach the pH ~ 8. Then CuCl_2_·2H_2_O (1 mol. equiv.) was added and the suspension was mixed for 30 min at 20 °C. The product was collected by the filtration under reduced pressure and washed first with methanol and later wih diethyl ether. The complex was left to dry overnight at 45 °C. Yield: 88% (77 mg), green solid. ESI-HRMS (CH_3_CN): m/z calcd for [M+H]^+^: 315.9396; found: 315.9398; IR selected bands (ATR): ν = 3095, 3073, 1458, 1415, 1194, 1164, 1148, 1089, 754, 708 cm^−1^; elemental analysis calcd (%) for C_10_H_8_CuN_2_O_2_S_2_: C 38.03, H 2.55, N 8.87; found C 37.63, H 2.23, N 8.66.

### Bis[1-hydroxypyridine-2(1***H***)-thionato-***S,O***]nickel(II) (Ni-Pth)

Ligand pyrithione (also 2-mercaptopyridine-*N*-oxide; 70 mg, 2 mol. equiv.) was dissolved in 5 mL of methanol. While stirring 1 M NaOH_(aq)_ was added dropwise to reach the pH ~ 8. Then Ni(CH_3_CO_2_)_2_·4H_2_O (1 mol. equiv.) was added and the suspension was mixed for 4.5 h at 20 °C. The product was collected by the filtration under reduced pressure and washed first with methanol and later with diethyl ether. The complex was left to dry overnight at 45 °C. Yield: 77% (66 mg), dark red solid. ^1^H NMR (500 MHz, CDCl_3_): *δ* = 8.01 (dd, 2H, *J* = 6.8, 0.7 Hz, Ar–*H*), 7.36 (dd, 2H, *J* = 8.4, 1.6 Hz, Ar–*H*), 7.13–7.07 (m, 2H, Ar–*H*), 6.78 (td, 2H, *J* = 6.8, 1.6 Hz, Ar–*H*); ESI-HRMS (CH_3_CN): m/z calcd for [M+H]^+^: 310.9453; found: 310.9450; IR selected bands (ATR): ν = 3095, 3067, 1460, 1184, 1141, 741, 712, 695, 629, 442 cm^−1^; elemental analysis calcd (%) for C_10_H_8_N_2_NiO_2_S_2_: C 38.62, H 2.59, N 9.01; found C 38.64, H 2.20, N 8.96.

### Crystal structure determinations

The crystal structures for Ru-Pth and Ru-Pth-Me were reported by Kljun et al.^17^ and Kladnik et al.^16^, respectively, whereas structures for Ni-Pth and Cu-Pth (*cis* and *trans*) were described by Chen et al.^12^, Niu et al.^40^ and Bond et al.^23^, respectively. Schemes showing crystal structures of mentioned compounds can be found in the SI (Figures S1–S3) and were taken from the CCDC database under deposition numbers 1444459, 1912502, 1200484, 862274 and 174803 for Ru-Pth, Ru-Pth-Me, Ni-Pth, *cis*-Cu-Pth and *trans*-Cu-Pth, respectively.

### Catalytic testing

The photocatalytic oxidation of styrene was carried out in the aqueous catalyst suspensions, containing styrene (10 mmol L^–1^) in acetonitrile as a co-solvent (typically 50 vol%). Neat catalysts (10 mg) were suspended in 50 mL acetonitrile and an aliquot of styrene (5 mL) was added to the suspension and mixed well (300 rpm). A light source was used in all photocatalytic oxidation experiments, a low-pressure mercury lamp (Heraeus, Strahler 150 W Electrical Power, 878 mm Length, 15 mm Diameter, ~ 2 W cm^−1^ power density). The spectral energy distribution of the lamp was shown in the SI, Figure S20. To understand the photoinduced oxidation reactions and the roles, played by metal complexes in photocatalytic process, neat reaction without the catalyst was used initially in different irradiation systems, i.e. the monochromatic ultraviolet with the emission line at 254 nm (UV), the UV–Vis with the range of 200–600 nm, and the near UV to the visible region with the range of 300–400 nm. The catalytic testing of the other prepared catalysts was performed under UV–Vis irradiation. The calculations of absorption coefficients were done using the method demonstrated by Su et al.^41^, and the latter was in agreement with the literature values. A characteristic absorption peak for styrene was located at 245 nm with the molar attenuation coefficients (ε) of 90 (245 nm). No absorption for styrene was found after the wavelength of 270 nm. The absorption peaks of styrene oxide and benzaldehyde were located at 282 and 275 nm, respectively, and all molar attenuation coefficient values were (several) orders of magnitude higher than that of benzene. The concentration of styrene, styrene oxide and benzaldehyde during and after an experiment was calculated using the mentioned molar attenuation coefficients. Sample aliquots were withdrawn by a 1 mL syringe intermittently during illumination and filtered through a filter (EMD Millipore) to remove catalyst particles. The main products obtained during this reaction are styrene oxide, benzaldehyde, benzoic acid, 1-phenylethane-1,2-diol and acetophenone (Electronic supplementary information, Figure S1). The preliminary identification and confirmation of the styrene, styrene oxide, benzaldehyde and other aromatic intermediates was done by using a high-performance liquid chromatography (HPLC, Agilent 1100 Series), equipped with the Waters XSelect CSHC18 column (the packing particle size of 3.5 µm and the column dimensions of 4.6 mm × 100 mm) at the temperature of 35 °C. Eluent/mobile phase solution was composed of acetonitrile (65 vol%) and water (35 vol%), while the flow rate of 1 mL min^–1^ was used. All the experiments were obtained in duplicate with a standard deviation of ± 1% and the carbon balances are ranged between 95 and 97%. The turnover frequency of the styrene was calculated using the moles of styrene converted per moles of metal per hour. The turnover number was calculated using the moles of styrene oxide formed per metal atom.

## Supplementary Information

Supplementary Information.
