# Supplementary material for: Pyrithione metal (Cu, Ni, Ru) complexes as photo-catalysts for styrene oxide production
Source: Sci Rep. 2021 Dec 10;11:23810. doi: 10.1038/s41598-021-03085-2 (PMC8664809; doi:10.1038/s41598-021-03085-2)
Supplement: Supplementary file 1 — Supplementary Information. [file 41598_2021_3085_MOESM1_ESM.docx]

**Supplementary Information**





**Figure S1:** Plausible oxidation products in the photocatalytic oxidation of styrene.


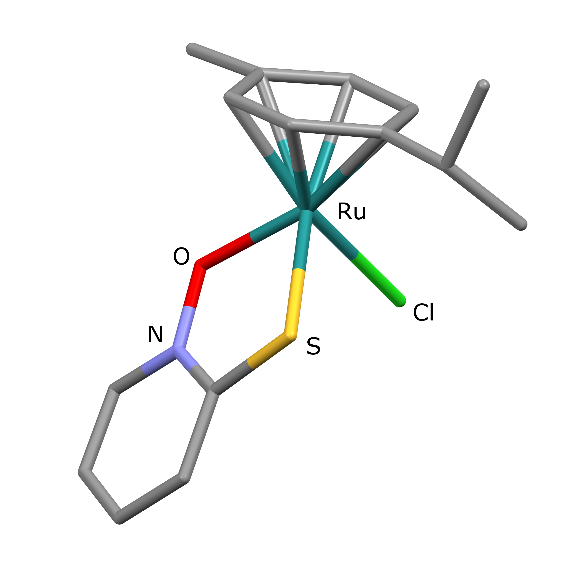

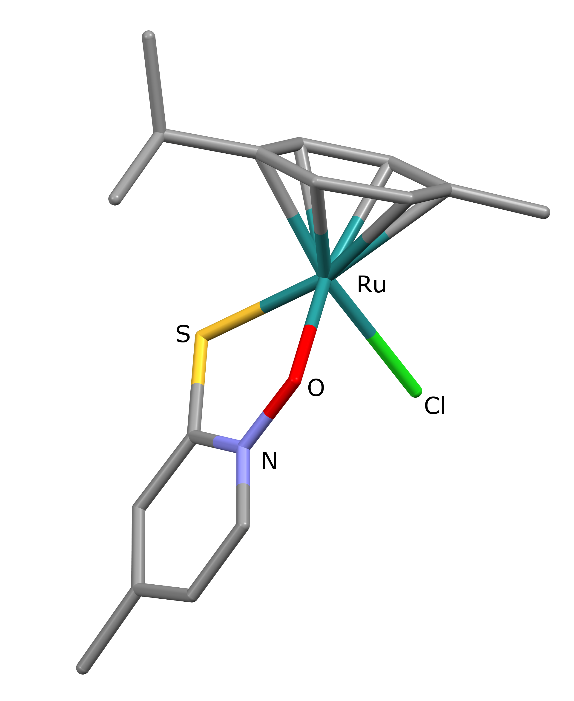


**Figure S2:** Crystal structure of the ruthenium catalysts Ru-Pth (left)^1^ and Ru-Pth-Me (right).^2^


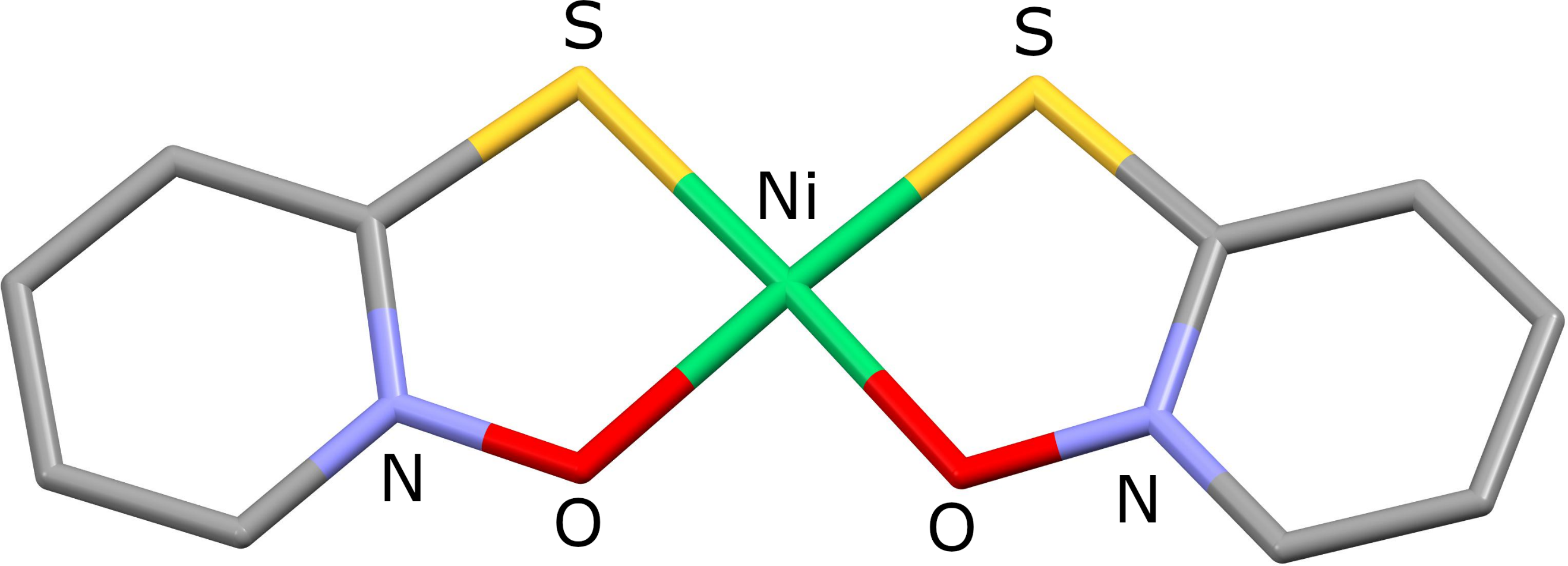


**Figure S3:** Crystal structure of the nickel catalyst Ni-Pth.^3^


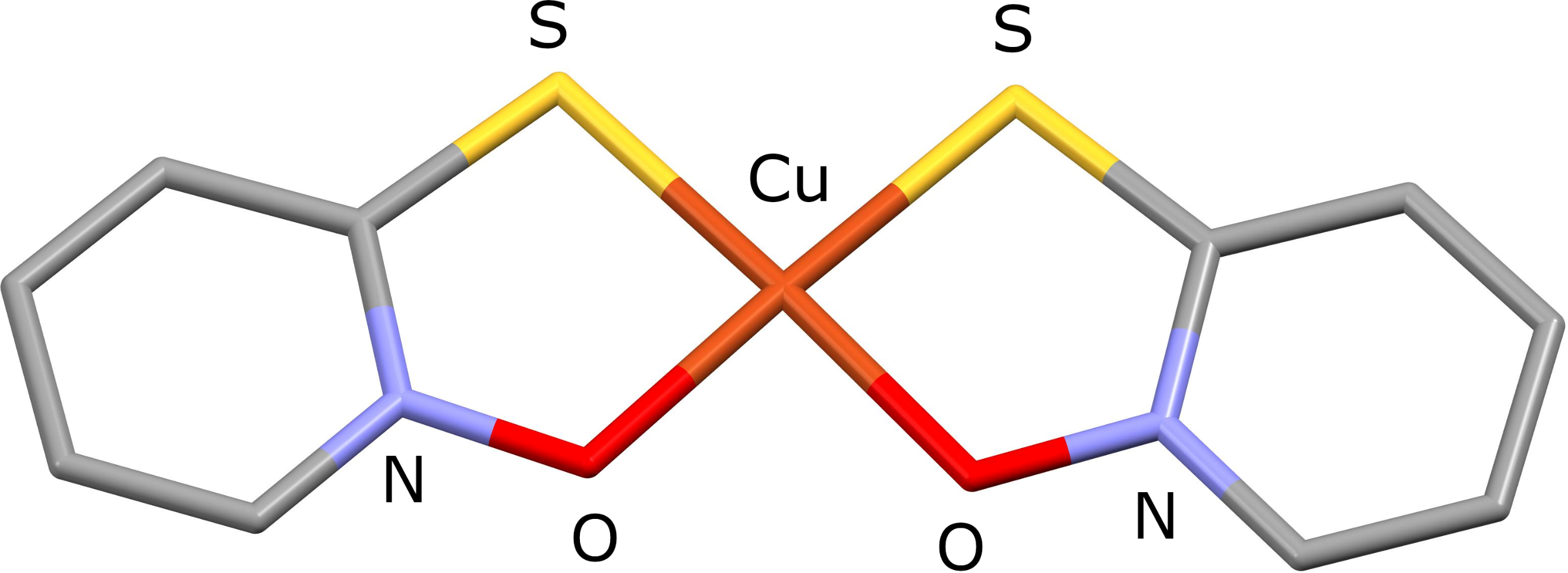


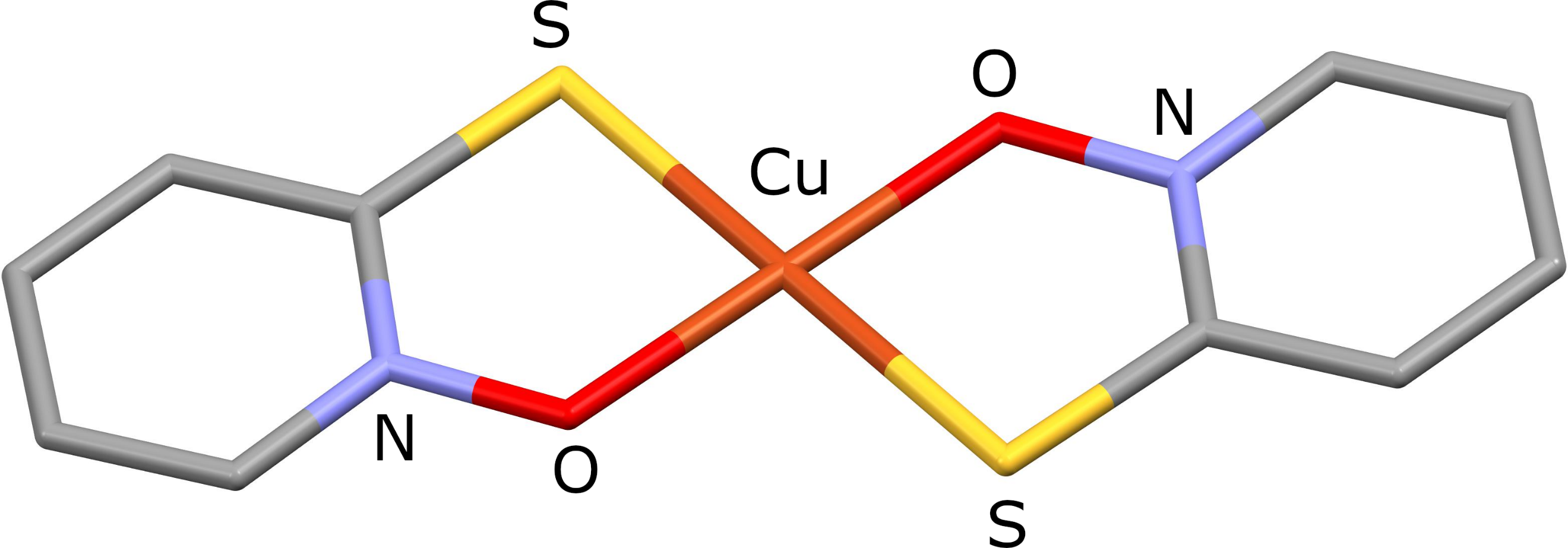


**Figure S4:** Crystal structure of the copper catalysts *cis*-Cu-Pth (top)^4^ and *trans*-Cu-Pth (bottom).^5^

**Figure S5:** IR spectrum of Pth.

**Figure S6:** IR spectrum of Pth-Me.

**Figure S7:** IR spectrum of [Ru(*p*-cymene)Cl_2_]_2_.

**Figure S8:** IR spectrum of Ru-Pth.

**Figure S9:** IR spectrum of Ru-Pth-Me.

**Figure S10:** IR spectrum of Cu-Pth.

**Figure S11:** IR spectrum of Ni-Pth.

**A**

**B**

**Figure S12:** Comparison of the IR spectra of the ligands Pth and Pth-Me, ruthenium precursor [Ru(*p*-cymene)Cl_2_]_2_ and ruthenium complexes Ru-Pth and Ru-Pth-Me (**A**) and of the ligand Pth and its Cu and Ni complexes (**B**).

When compared IR spectra of the investigated compounds, these are similar for the ruthenium pair of compounds Ru-Pth and Ru-Pth-Me as well as for Ni-Pth and Cu-Pth pair. In case of the ruthenium complexes aromatic C–H stretching vibrations between 3100-3000 cm^-1^ can be found belonging to the *p*-cymene and pyrithionato aromatic rings. Additionally, there are also C–H stretching vibrations between 3000-2800 cm^-1^ characteristic for methyl and isopropyl groups of *p*-cymene and for methyl group of the *O,S*-ligand in case of Ru-Pth-Me. Fingerprint regions of the compounds represent complicated series of absorptions. It is obvious that some bands are shifted upon binding of the pyrithione ligands to the metal ion, but complete assignment is not possible. Still, some peaks can be approximately identified. Ru-Pth and Ru-Pth-Me possess absorption bands at 1544 (free ligand Pth at 1564 cm^-1^) and 1537 cm^-1^ (free ligand Pth-Me at 1549 cm^-1^), respectively, which could belong to the *N*-oxide group of the pyrithione moiety. Besides, some other bands of the ligand Pth have been shifted in the Ru complexes. When compared the spectra of Ni-Pth, Cu-Pth and the free ligand Pth only aromatic C–H stretching vibrations between 3100-3000 cm^-1^ exist, indicating the presence of pyrithione ligand in the absence of *p*-cymene ring. In comparison to the absorption bands of the ligand Pth in the interval between 3105-3026, this interval in Cu-Pth is between 3095-3029 or 3095-3030 for Ni-Pth. Absorption band of the free Pth at 1564 cm^-1^ might again presented *N*-oxide group at 1546 and 1548 cm^-1^ of Cu-Pth and Ni-Pth complexes, respectively.^6^ Obtained IR spectra are in a good agreement with the one of Pth sodium salt and its zinc complex.^7^

**Figure S13:** Kubelka–Munk function graph for Ru-Pth complex.

**Table S1:** Selective photocatalytic oxidation of styrene oxide over metal pyrithione complexes.

*Reaction conditions: Catalyst to styrene oxide ratio of (1 : 100); styrene oxide : H_2_O_2_ (1 : 1); time: 3 h; solvent: acetonitrile; temperature: 60 °C*

| **Catalyst** | **Styrene Oxide Conversion (%)** | **Selectivity (mol %)** | | |
| --- | --- | --- | --- | --- |
|  |  | **Benzaldehyde** | **Benzoic Acid** | **Acetophenone** |
| Ru-Pth | 59.6 | 46.3 | 22.5 | 14.7 |
| Ru-Pth-Me | 35.8 | 41.8 | 28.5 | 24.1 |
| Ni-Pth | 31.4 | 52.3 | 25.6 | 21.8 |
| Cu-Pth | 48.2 | 54.2 | 23.7 | 15.7 |

**Table S2:** Selective photocatalytic oxidation of styrene oxide over metal pyrithione complexes.

*Reaction conditions: Catalyst to styrene oxide ratio of (1 : 100); styrene oxide : H_2_O_2_ (1 : 1); time: 3 h; solvent: acetonitrile; temperature: 60 °C*

| **Catalyst** | **Benzaldehyde conversion (%)** | **Selectivity (mol %)** | | |
| --- | --- | --- | --- | --- |
|  |  |  | **Benzoic Acid** |  |
| Ru-Pth | 45.2 |  | 96.3 |  |
| Ru-Pth-Me | 38.6 |  | 94.7 |  |
| Ni-Pth | 26.7 |  | 95.7 |  |
| Cu-Pth | 35.7 |  | 97.8 |  |


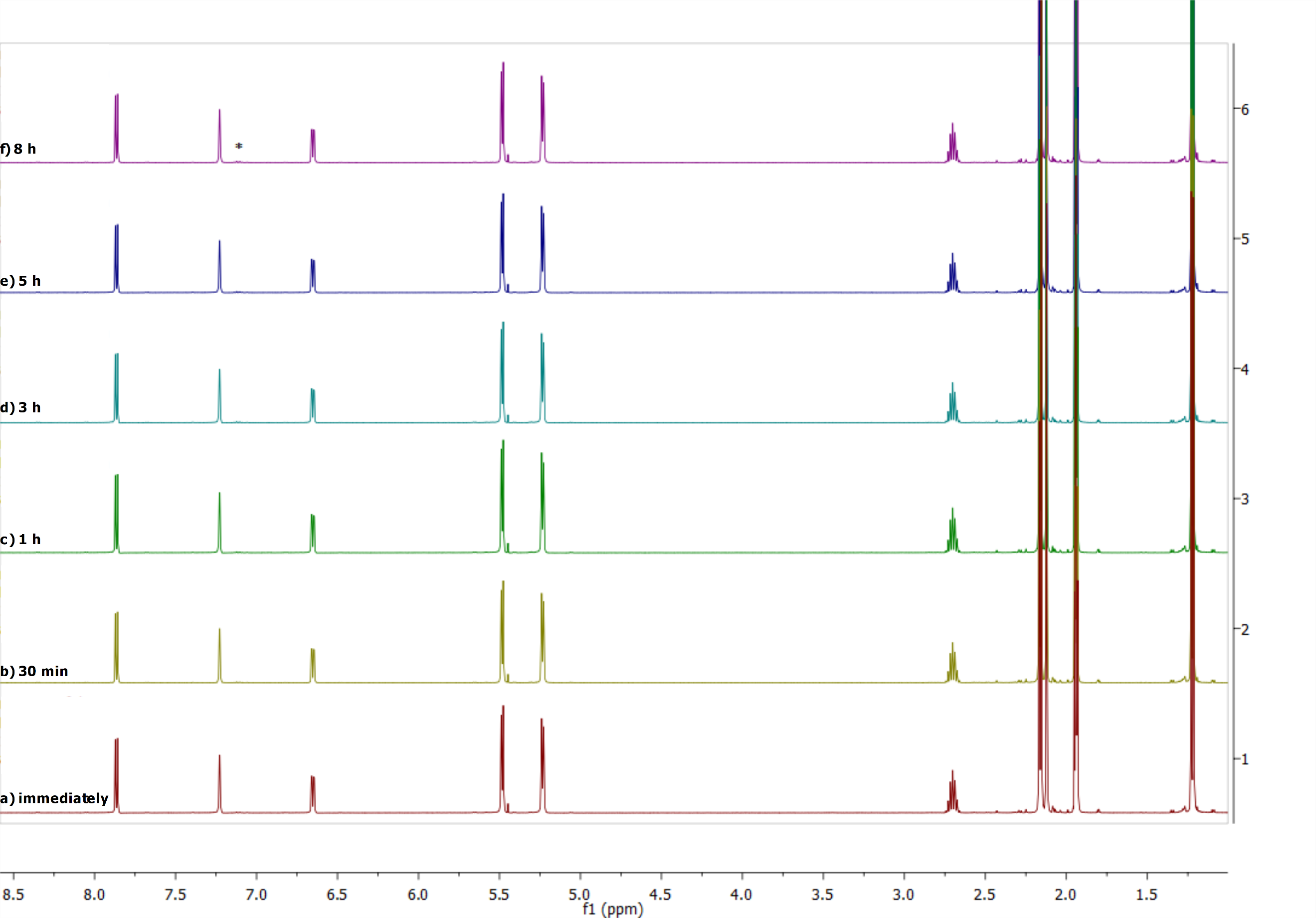


**Figure S14:** Spectra of Ru-Pth-Me in acetonitrile-d_3_ followed by ^1^H NMR spectroscopy at different time points. The release of the *p*-cymene is marked with an asterisk (*).


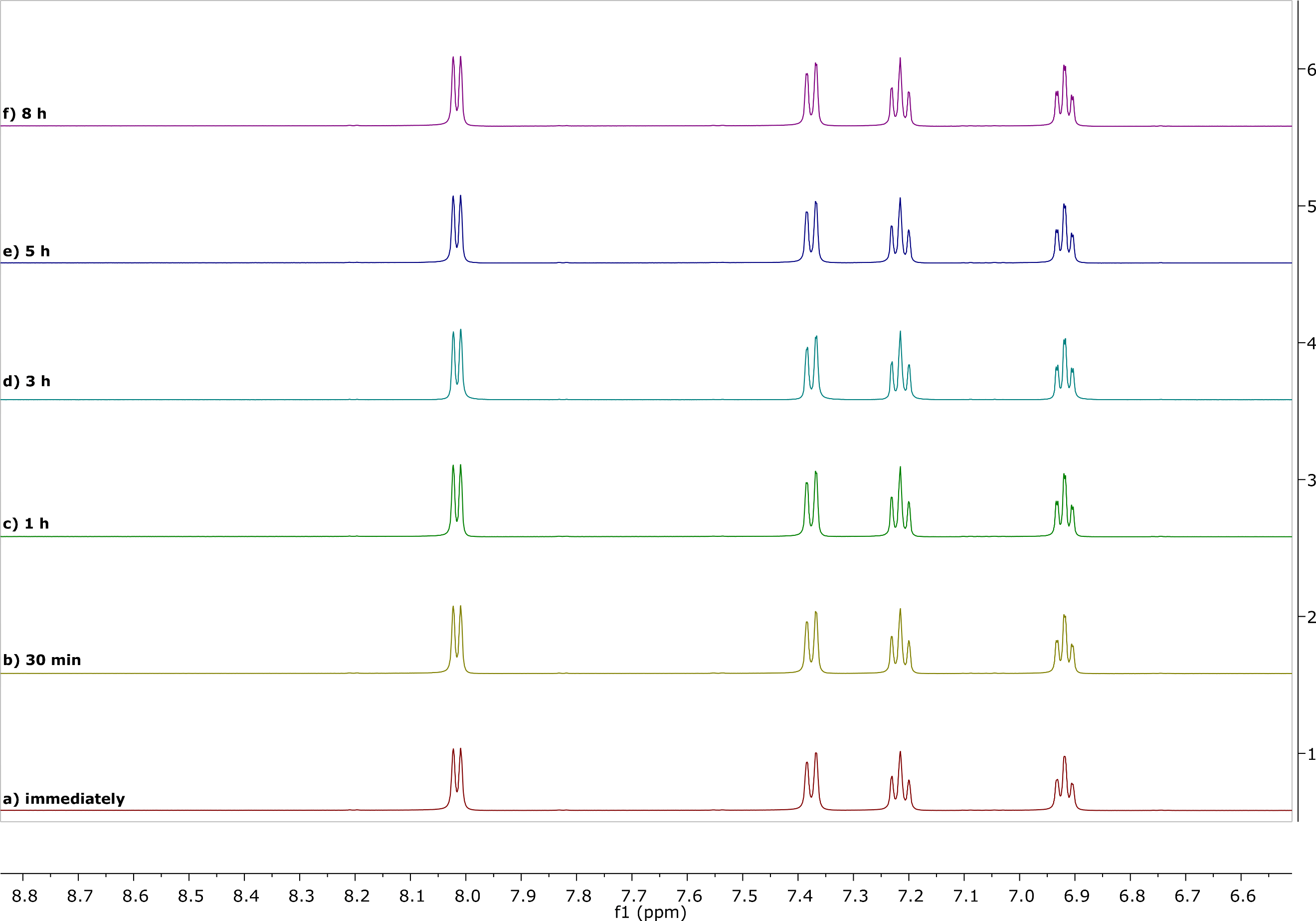


**Figure S15:** Spectra of Ni-Pth in acetonitrile-d_3_ followed by ^1^H NMR spectroscopy at different time points.





**Figure S16:** A comparison of the TG curves of the prepared complexes in a temperature range from 25 °C and 400 °C. Onset decomposition temperature is given at the beginning of thermal decomposition of each complex.

**
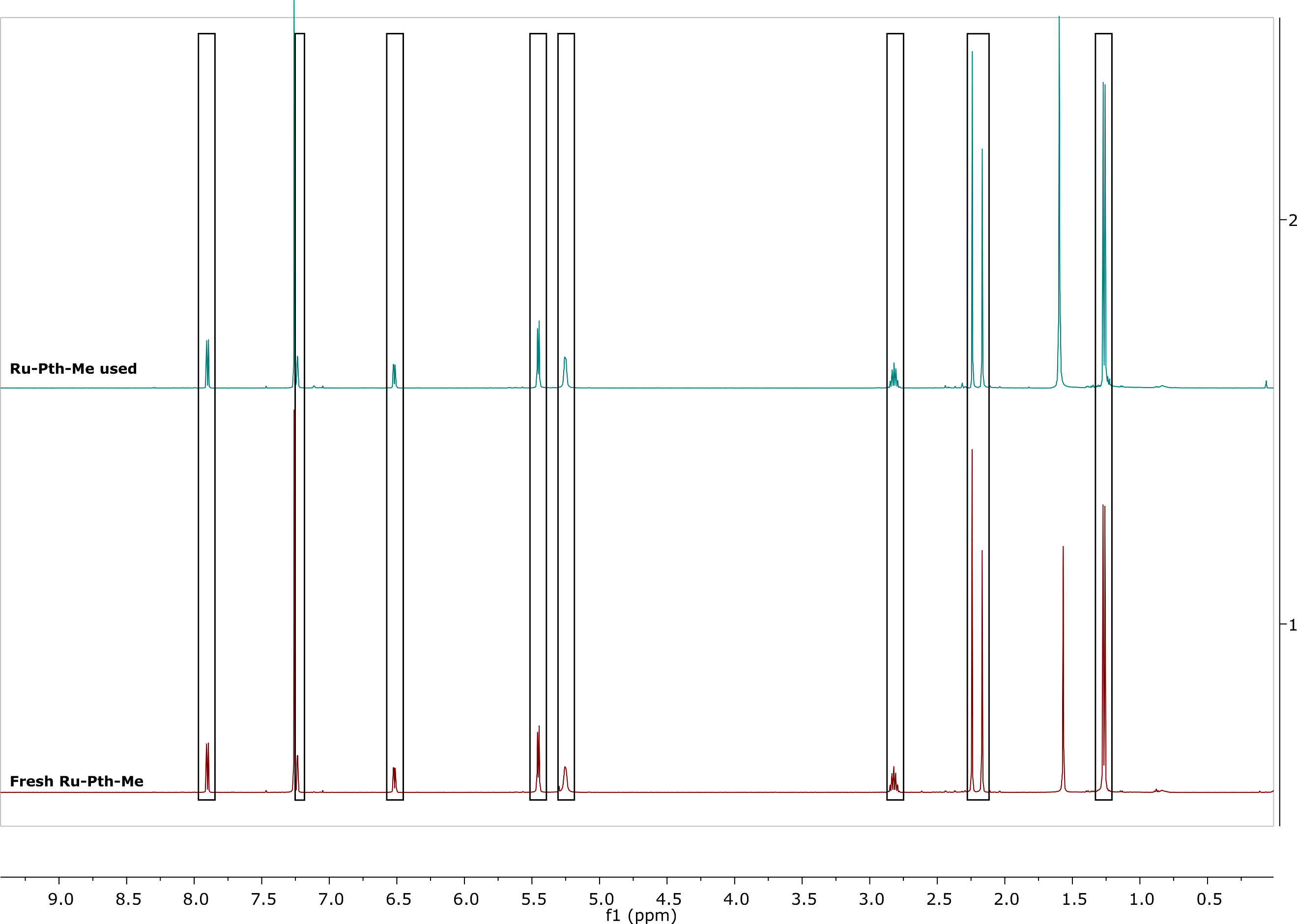
**

**Figure S17:** ^1^H NMR spectra in CDCl_3_ of the initial Ru-Pth-Me complex before catalysis (fresh Ru-Pth-Me) and after (Ru-Pth-Me used) under optimized conditions (catalyst: styrene (1 : 100); styrene: H_2_O_2_ (1 : 1); time: 3 h; solvent: acetonitrile; temperature: 70 °C.

**
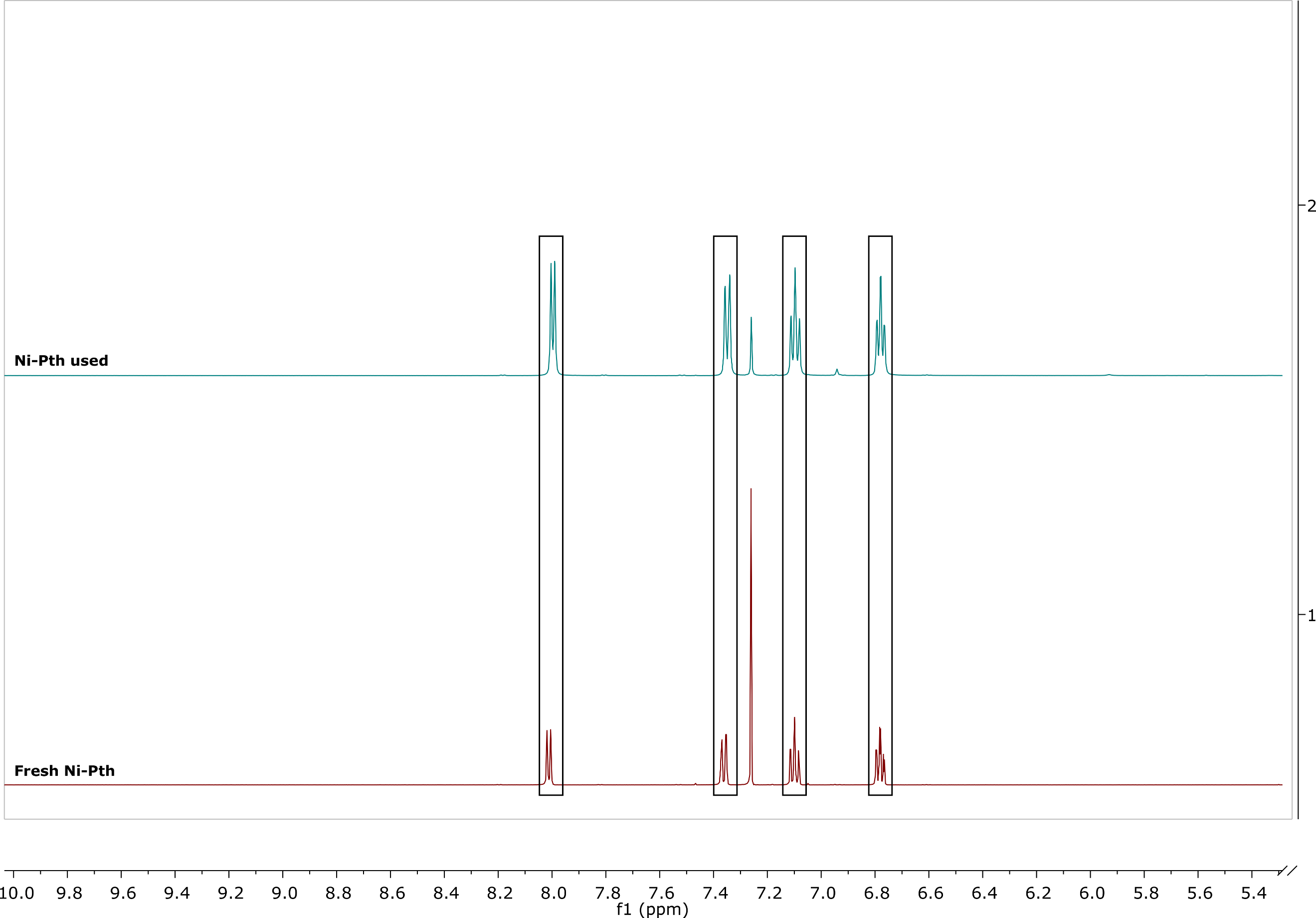
**

**Figure S18:** ^1^H NMR spectra in CDCl_3_ of the initial Ni-Pth complex before catalysis (fresh Ni-Pth) and after (Ni-Pth used) under optimized conditions (catalyst: styrene (1 : 100); styrene: H_2_O_2_ (1 : 1); time: 3 h; solvent: acetonitrile; temperature: 70 °C.


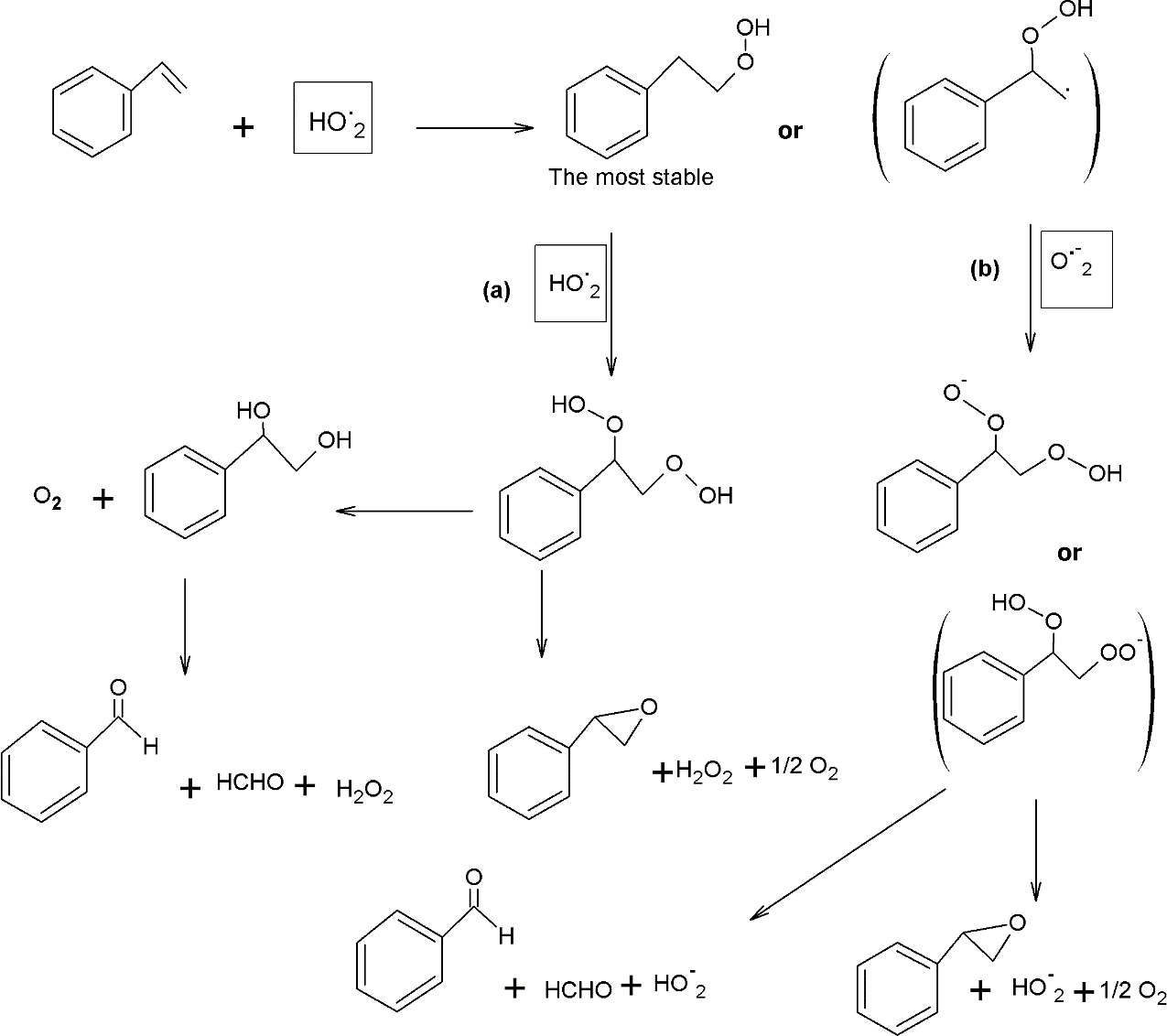


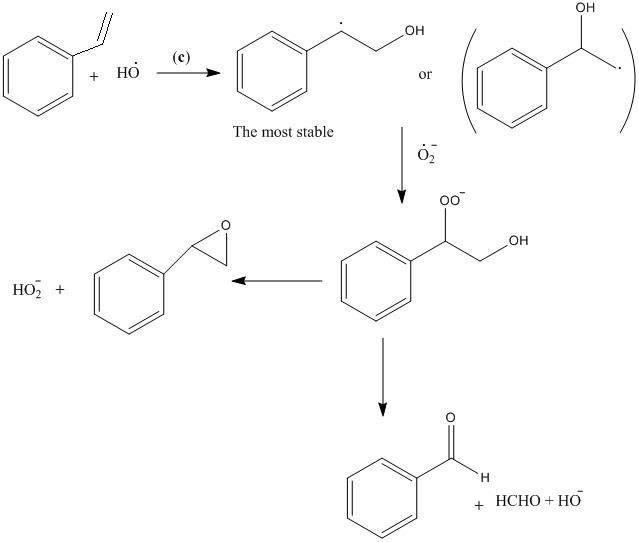


**Figure S19**: proposition of the chemical pathways of the reaction of styrene with two HO2° radicals (mechanism a), or one HO° and O2°^-^ (mechanism b) or one HO2° and one O2° (mechanism c)


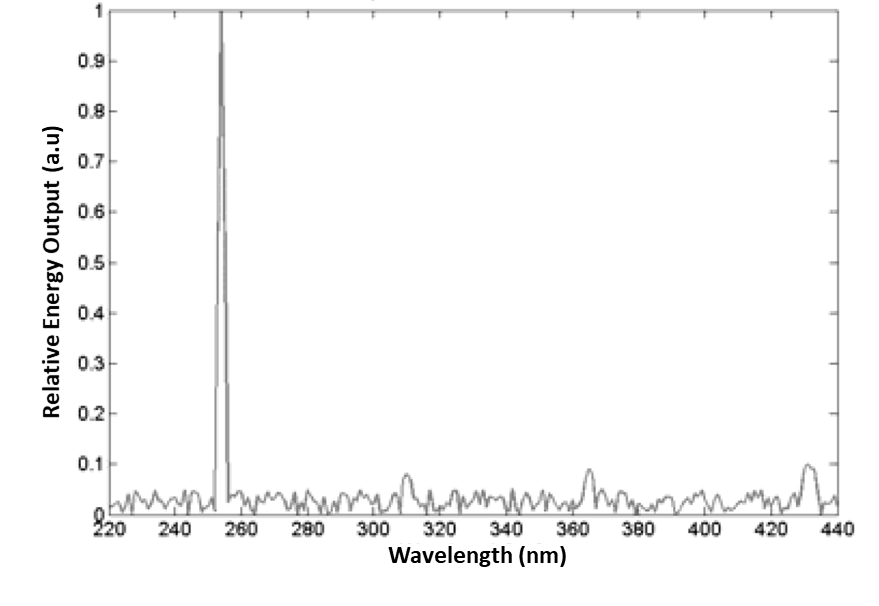


**Figure S20:** Spectral output of Low pressure mercury lamp

References:

1 Kljun, J. *et al.* Pyrithione-based ruthenium complexes as inhibitors of aldo-keto reductase 1C enzymes and anticancer agents. *Dalton Trans.* **45**, 11791-11800 (2016).

2 Kladnik, J. *et al.* Towards Identification of Essential Structural Elements of Organoruthenium(II)-Pyrithionato Complexes for Anticancer Activity. *Chem. Eur. J.* **25**, 14169–14182 (2019).

3 Chen, X., Hu, Y., Wu, D., Weng, L. & Kang, B. Syntheses and electrochemistry of some transition metal complexes with 2-mercaptopyridine N-oxide and crystal structure of bis(2-mercaptopyridine N-oxide)nickel(II). *Polyhedron* **10**, 2651-2657, doi:10.1016/s0277-5387(00)86163-x (1991).

4 Niu, D. Z., Yao, L., Min, X. & Zou, H. Crystal structure of *cis*-bis[1-hydroxypyridine-2(1*H*)-thionato-*S,O*]copper(II), Cu(C_5_H_4_NOS)_2_. *Z. Kristallogr. NCS* **226**, 527-528, doi:10.1524/ncrs.2011.0234 (2011).

5 Bond, A. D., Feeder, N., Teat, S. J. & Jones, W. Bis[1-hydroxypyridine-2(1*H*)-thionato-*S,O*]copper(II). *Acta Crystallogr. C* **57**, 1157-1158, doi:10.1107/s0108270101012306 (2001).

6 Colthup, N. B., Daly, L. H. & Wiberley, S. E. *Introduction to infrared and raman spectroscopy*. 3rd ed. edn, (Academic Press, 1990).

7 Kalantar, T. H. *et al.* A green synthesis of bis 1-(hydroxy-κ*O*)-2(1*H*)-pyridinethionato-κ*S*^2^]-(*T*-4)-zinc (zinc pyrithione) nanoparticles via mechanochemical milling. *J. Exp. Nanosci.* **11**, 138-147, doi:10.1080/17458080.2015.1036323 (2016).
